# Paecilomycone Inhibits Quorum Sensing in Gram-Negative Bacteria

**DOI:** 10.1128/spectrum.05097-22

**Published:** 2023-03-15

**Authors:** Wouter A. G. Beenker, Jelmer Hoeksma, Marie Bannier-Hélaouët, Hans Clevers, Jeroen den Hertog

**Affiliations:** a Hubrecht Institute-KNAW and University Medical Center Utrecht, Utrecht, The Netherlands; b Oncode Institute, Hubrecht Institute-KNAW and University Medical Center, Utrecht, The Netherlands; c Institute Biology Leiden, Leiden University, Leiden, The Netherlands; University of Nebraska Medical Center

**Keywords:** paecilomycone, *P. aeruginosa*, quorum sensing, phenazines, 4-hydroxy-2-alkylquinolines, *Pseudomonas aeruginosa*

## Abstract

Pseudomonas aeruginosa is an opportunistic pathogen that causes major health care concerns due to its virulence and high intrinsic resistance to antimicrobial agents. Therefore, new treatments are greatly needed. An interesting approach is to target quorum sensing (QS). QS regulates the production of a wide variety of virulence factors and biofilm formation in P. aeruginosa. This study describes the identification of paecilomycone as an inhibitor of QS in both Chromobacterium violaceum and P. aeruginosa. Paecilomycone strongly inhibited the production of virulence factors in P. aeruginosa, including various phenazines, and biofilm formation. In search of the working mechanism, we found that paecilomycone inhibited the production of 4-hydroxy-2-heptylquinoline (HHQ) and 3,4-dihydroxy-2-heptylquinoline (PQS), but not 2′-aminoacetophenone (2-AA). Therefore, we suggest that paecilomycone affects parts of QS in P. aeruginosa by targeting the PqsBC complex and alternative targets or alters processes that influence the enzymatic activity of the PqsBC complex. The toxicity of paecilomycone toward eukaryotic cells and organisms was low, making it an interesting lead for further clinical research.

**IMPORTANCE** Antibiotics are becoming less effective against bacterial infections due to the evolution of resistance among bacteria. Pseudomonas aeruginosa is a Gram-negative pathogen that causes major health care concerns and is difficult to treat due to its high intrinsic resistance to antimicrobial agents. Therefore, new targets are needed, and an interesting approach is to target quorum sensing (QS). QS is the communication system in bacteria that regulates multiple pathways, including the production of virulence factors and biofilm formation, which leads to high toxicity in the host and low sensitivity to antibiotics, respectively. We found a compound, named paecilomycone, that inhibited biofilm formation and the production of various virulence factors in P. aeruginosa. The toxicity of paecilomycone toward eukaryotic cells and organisms was low, making it an interesting lead for further clinical research.

## INTRODUCTION

Pseudomonas aeruginosa is a Gram-negative pathogen that causes nosocomial infections in immunocompromised patients. It is involved in a variety of acute and chronic infections, including urinary tract infections, burns or wound infections, and respiratory diseases, like cystic fibrosis (CF) (1, 2). P. aeruginosa has a high intrinsic resistance due to the low permeability of its outer membrane, the large amount of efflux pumps, and the capability to form biofilms. Therefore, P. aeruginosa infections are difficult to treat (1, 3). Once established in the lung, P. aeruginosa infections often become chronic, contributing to morbidity and mortality of the patients (4, 5). Therefore, new treatments against P. aeruginosa infections are highly needed, and quorum sensing (QS) as target might be an interesting approach.

QS in bacteria is a communication system that involves changes in gene expression in response to cell density. In Gram-negative bacteria, the QS systems are very similar, with small modifications. These systems contain homologues of a LuxI-type synthase that produces acylated homoserine lactones (AHLs) that are specific to that bacterial strain. When the number of bacteria increases, the concentration of AHL increases. The AHL will bind to its cognate LuxR-type receptor, which subsequently binds to the promoter of QS target genes and alters gene expression (6–8).

Chromobacterium violaceum contains a single LuxI/R-type QS network, with CviI as a synthase and CviR as a receptor. In contrast, P. aeruginosa has three different QS systems. Two of these systems are typical Gram-negative bacterial LuxI/R-type QS systems, the *las*-encoded system and the *rhl*-encoded system. The third system in P. aeruginosa is a unique system based on 4-hydroxy-2-alkylquinolines (HAQs) that are synthesized by the enzymes of the *pqsABCDE* operon and PqsH. Among these HAQs are 3,4-dihydroxy-2-heptylquinoline (PQS) and its precursor, 4-hydroxy-2-heptylquinoline (HHQ), which can bind to PqsR (also called MvfR) (9–11). Via positive- and negative-feedback mechanisms, these three QS systems are highly interconnected. The Las system is often placed on top of this hierarchy due to positive regulation of the Rhl and PQS system (10). However, this hierarchy is flexible and can shift under certain growth conditions (12). Some papers report 2-(2-hydroxylphenyl)-thiazole-4-carbaldehyde (IQS) as a fourth QS system in P. aeruginosa. However, this is highly debatable, and therefore IQS is excluded from this paper (13).

The three QS systems in P. aeruginosa play a major role in the production of virulence factors and biofilm formation. Therefore, inhibition of QS would lead to decreased production of virulence factors, including pyocyanin, rhamnolipids, and elastase, and a concomitant decrease in biofilm formation (14, 15). This way, QS inhibitors might reduce toxicity of the bacteria toward the host, due to lower production of toxic virulence factors, and at the same time higher susceptibility to conventional antibiotics, due to a weakened biofilm (16, 17). Promising effects of QS inhibition have already been shown by a wide variety of QS inhibitors from various sources, including quercetin from oak (18), ajoene from garlic (16), and furanones from algae (19).

In a previous study from our lab, we reported the search for novel QS inhibitors among secondary metabolites of 10,207 strains of fungi. One of the QS inhibitors we identified was desmethyl-gregatin A, produced by Aspergillus allahabadii (20). Here, we report identification of another active fraction from *A. allahabadii*, containing paecilomycone, which has QS inhibitor activity against C. violaceum and P. aeruginosa. Paecilomycone treatment showed a great reduction in biofilm formation, phenazine production, and HAQ synthesis. In addition, toxic effects appeared to be low, especially in complex systems, making paecilomycone an interesting molecule for further development for clinical use.

## RESULTS

### Identification of paecilomycone.

For the purification of paecilomycone, we fractionated the growth medium containing Aspergillus allahabadii secondary metabolites by using a preparative high-performance liquid chromatography (HPLC) system, using trifluoroacetic acid (TFA) as modifier (see Fig. S1A in the supplemental material). The fractions were tested for quorum sensing (QS) inhibitor activity using C. violaceum as the reporter bacterium and violacein production as a readout. C. violaceum produces a purple pigment, named violacein, upon activation of this QS network, making it a good reporter to search for novel QS inhibitors (21). In a previous study, we reported desmethyl-gregatin A as active compound from this fungus (20). However, another fraction, fraction 20, also showed inhibition of violacein production.

In this study, we purified the active compound from fraction 20 by refractionating the active fraction by preparative HPLC using ammonium acetate as modifier (Fig. S1B). Again, the fractions were tested, and fraction 1 showed QS inhibitor activity. The yield of 3 L Aspergillus allahabadii supernatant was on average 3.5 mg of dried fraction 1.

**FIG 1:**
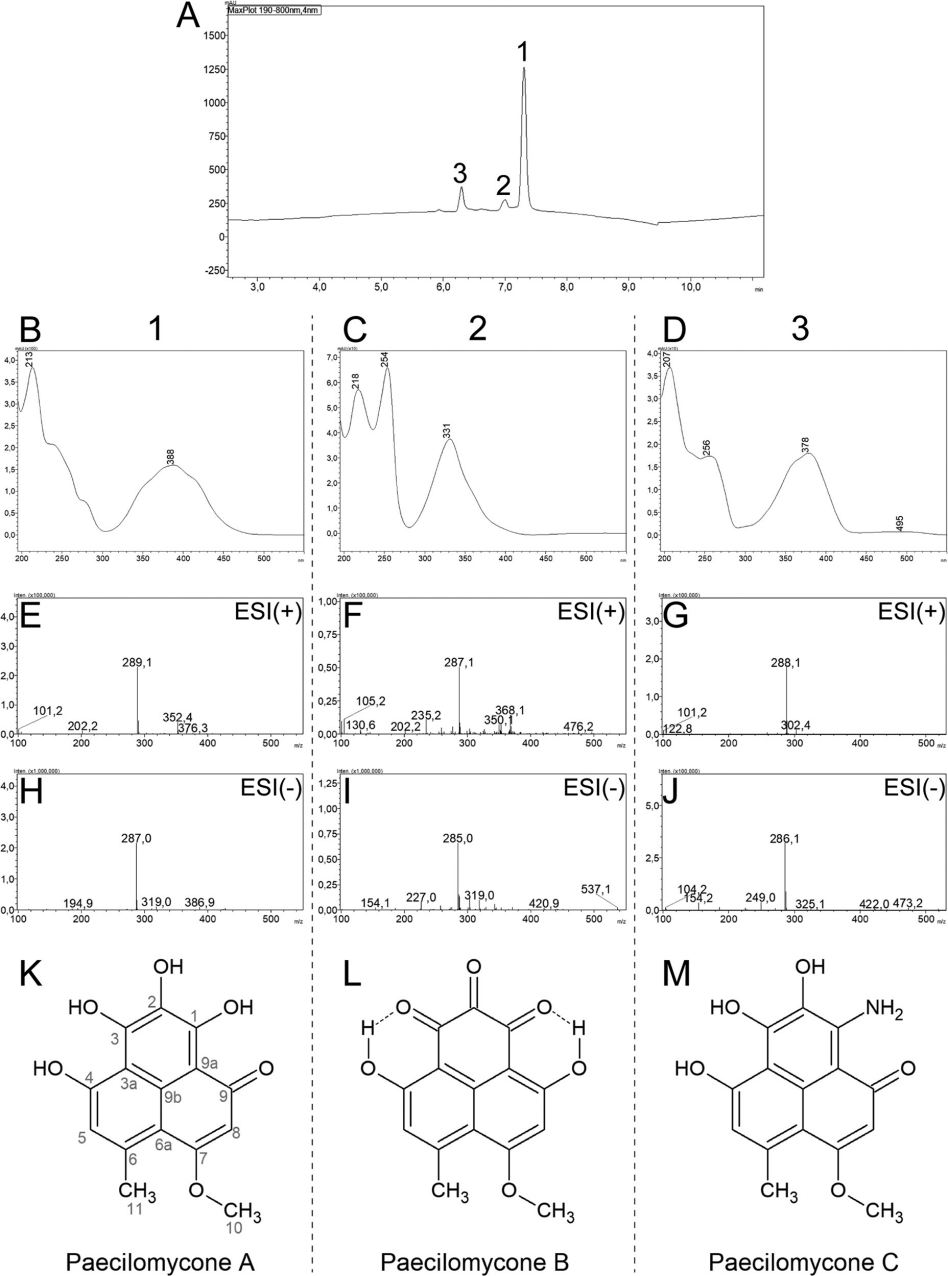
Identification of paecilomycone. (A) Analytical HPLC (aHPLC) spectrogram of paecilomycone showing 3 peaks. (B to D) UV-Vis chromatogram of (B) peak 1, (C) peak 2, and (D) peak 3; (E to G) LC-MS electrospray ionization (ESI^+^ [positive-mode]) spectrogram of (E) peak 1, (F) peak 2, and (G) peak 3; (H to J) LC-MS ESI^−^ (negative-mode) spectrogram of (H) peak 1, (I) peak 2, and (J) peak 3; (K to M) chemical structures of paecilomycones belonging to (K) peak 1, (L) peak 2, and (M) peak 3.

When running fraction 1 on analytical HPLC, we found three peaks (Fig. 1A). Peak 1 was the largest and showed a characteristic UV-visible (UV-Vis) spectrum [**213(100)**, 238sh, 277sh, 388(40)] and *m/z* values of 289.1 [M+H]^+^ and 287.0 [M−H]^−^, suggesting a nominal mass of 288 (Fig. 1B, E, and H). In addition, high-resolution mass spectrometry measured a mass of 289.5937, giving a calculated monoisotopic mass of 289.0692 [M+H]^+^, with a molecular formula prediction of C_15_H_12_O_6_ (Fig. S2). This UV-Vis spectrum and mass showed similarities to paecilomycone A (22). Therefore, the nuclear magnetic resonance (NMR) data were compared to the published data of paecilomycone A (Table 1; Fig. S3).

**TABLE 1:** ^13^C- and ^1^H-NMR spectral data of paecilomycone compared to those from Lu et al. in DMSO-*d_6_*

Position	Spectrum from:			
	Our data		Data from Lu et al.^*a*^	
	^13^C	^1^H	^13^C	^1^H
1	Not detected		166.8	
2	Not detected		131.7	
3	Not detected		172	
3a	105.96		105.4	
4	Not detected		162.2	
5	117.05	6.57 (s, 1H)	117.3	6.82 (s, 1H)
6	144.08		144.9	
6a	109.67		111.6	
7	165.97		166.8	
8	97.13	6.30 (s, 1H)	97.4	6.51 (s, 1H)
9	173.38		172.3	
9a	102.83		103.3	
9b	Not detected		127.8	
10	56.33	3.89 (s, 3H)	56.7	4,00 (s, 3H)
11	25.97	2.65 (s, 3H)	25.8	2.74 (s, 3H)

a See reference 22 for details.

Whereas not all carbons were detected, the NMR data, like the UV-Vis and liquid chromatography-mass spectrometry (LC-MS) data, showed high similarities to the data of Lu et al. (22). For further validation, the heteronuclear multiple-bond correlation (HMBC) data were compared (Fig. S4). The HMBC data showed the same correlations as those described before by Lu et al. (22), validating that this compound is indeed paecilomycone A (Fig. 1K).

However, next to paecilomycone A, two other small peaks were observed in the analytical HPLC chromatogram (Fig. 1). Peak 2 showed a UV-Vis spectrum [218(90), **254(100)**, 331(50)] and *m/z* values of 287.1 [M+H]^+^ and 285.0 [MH]^−^, suggesting a nominal mass of 286, corresponding to paecilomycone B (Fig. 1C, F, I, and L) (22). Peak 3 showed a UV-Vis [**207(100)**, 259 (50), 378 (50)] and *m/z* values of 288.1 [M+H]^+^ and 286.1 [MH]^−^, suggesting a nominal mass of 287, corresponding to paecilomycone C (Fig. 1D, G, J, and M) (22). The ratio of the various paecilomycones was ~85:5:10 (paecilomycone A/B/C, respectively), when dissolved in acetonitrile-water with 0.1% TFA. Using this setup, it was not possible to separate the various paecilomycones further. Therefore, the combination of the three paecilomycones will be referred to as paecilomycone, unless stated otherwise. In conclusion, the fraction of Aspergillus allahabadii with QS inhibitor activity contained paecilomycone.

### Paecilomycone shows QS inhibitor activity in C. violaceum.

**FIG 2:**
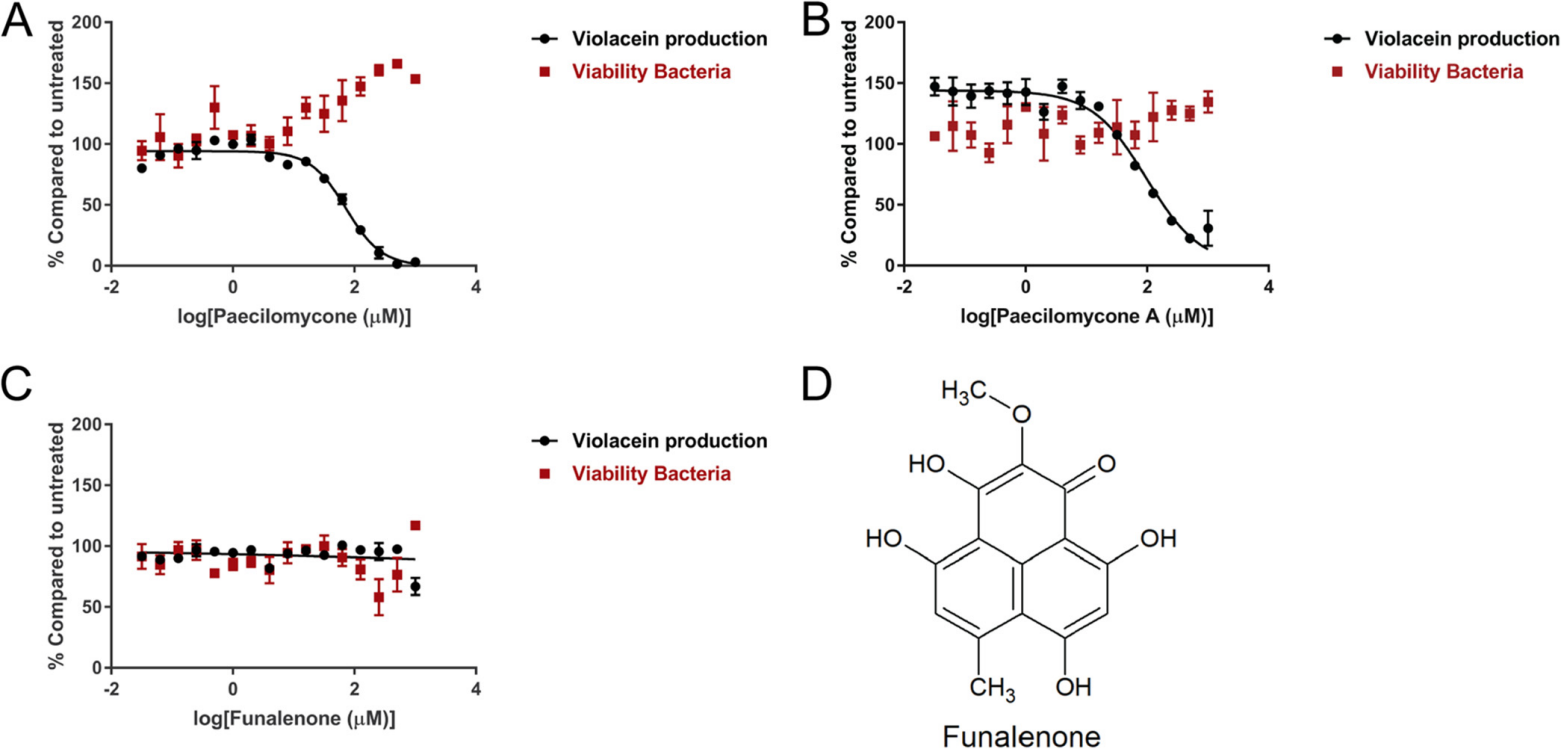
Quorum sensing inhibition by paecilomycone using C. violaceum as a reporter bacterium. (A to C) Violacein production and viability of C. violaceum after treatment with (A) paecilomycone, (B) a paecilomycone fraction lacking paecilomycone C, and (C) funalenone; (D) structure of funalenone. Experiments were done in triplicate, and error bars represent the standard error of the mean (SEM).

Paecilomycone strongly inhibited violacein production in C. violaceum, with an 50% inhibitory concentration (IC_50_) of 72.5 μM (Fig. 2A). Viability was measured in parallel to distinguish between effects on QS and effects on bacterial growth that could affect violacein production. No toxic effects of paecilomycone were detected on bacteria at these concentrations. Therefore, paecilomycone was a potent QS inhibitor at concentrations that do not affect cell viability.

Whereas the three forms of paecilomycone could not be separated further with the two-step purification we used, we found that, by using TFA instead of ammonium acetate in the second purification step, the amine group was not incorporated in the compound and paecilomycone C was not present in the mixture (Fig. S5). The fraction lacking paecilomycone C still contained paecilomycone A, a small amount of paecilomycone B (A/B ratio of 91:9), and an unidentified peak. This fraction showed a concentration-dependent effect on violacein production, with a similar IC_50_ to paecilomycone (IC_50_ = 96.5 μM) (Fig. 2B). Toxic effects on the viability of C. violaceum were not detected. This suggests that the most abundant paecilomycone, A, was responsible for most of the QS inhibitor effect of paecilomycone.

Next, we tested the effect of funalenone on QS inhibition. Funalenone is structurally highly related to paecilomycone A with only the position of the methoxy group being different (cf. Fig. 2D and Fig. 1K). Interestingly, this small difference in structure resulted in a big difference in activity since we did not find inhibition in the production of violacein after funalenone treatment up to a concentration of 1 mM (Fig. 2C).

Taken together, paecilomycone A and potentially paecilomycone B, but not the highly related funalenone, had QS inhibitor activity in C. violaceum.

### Paecilomycone shows QS inhibitor activity in P. aeruginosa.

**FIG 3:**
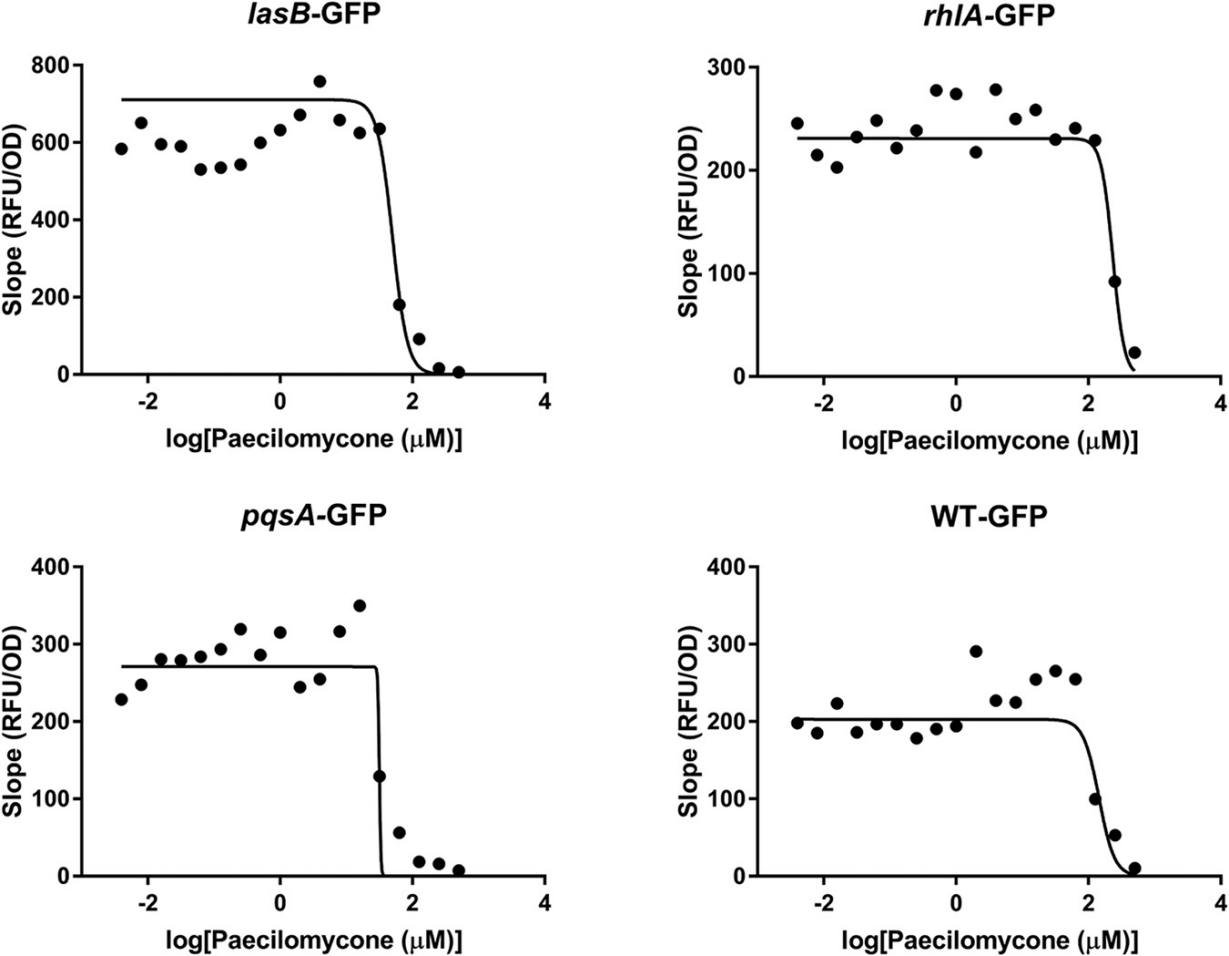
Quorum sensing inhibition in P. aeruginosa PAO1 reporter strains after paecilomycone treatment. The effect of paecilomycone was tested using *lasB*-GFP, *rhlA-*GFP, and *pqsA-*GFP reporters. In addition, the effect on the WT-GFP strain as a control was tested. The maximum slope of relative fluorescence units (RFU), normalized by growth, was plotted and used to calculate the IC_50_. Experiments were done three times in triplicate; the mean RFU/OD of a representative experiment was plotted.

To test the potential of paecilomycone as a QS inhibitor in more clinically relevant bacteria, the effect on various P. aeruginosa QS reporter strains was tested. P. aeruginosa PAO1 reporter strains were used that express green fluorescent protein (GFP) when the QS pathway is activated, including the *lasB-*GFP, *rhlA*-GFP, and *pqsA-*GFP strains. A wild-type strain that constitutively expressed GFP (WT-GFP) was used as control. GFP expression was normalized to the growth of the bacteria, and the IC_50_ was calculated by plotting the maximum slope of GFP expression/bacterial growth (Fig. 3).

Paecilomycone inhibited GFP expression in the *lasB-*GFP reporter and *pqsA-*GFP reporter strains, with IC_50_s of 49.8 μM and 31.5 μM, respectively (Fig. 3), which ares 3- to 4.5-fold lower than in control WT-GFP bacteria (IC_50_ = 143.2 μM), respectively. Concentrations of paecilomycone above 125 μM affected growth of P. aeruginosa, explaining the effect observed in WT-GFP bacteria (Fig. S6). The *rhlA*-GFP reporter strain was not sensitive to paecilomycone, with an apparent IC_50_ of 233.8 μM, which was actually higher than that of WT-GFP. We also tested the effect of funalenone on QS inhibition in PAO1. Funalenone did not show an inhibitory effect in any of the reporter strains (Fig. S7), indicating that funalenone did not inhibit QS in P. aeruginosa.

These results indicate that various, but not all, QS pathways were inhibited by paecilomycone—but not by the highly related funalenone—in clinically relevant P. aeruginosa (PAO1) bacteria, with paecilomycone showing the strongest effect on the *pqs*A-GFP reporter strain.

### Paecilomycone inhibits biofilm formation in P. aeruginosa.

**FIG 4:**
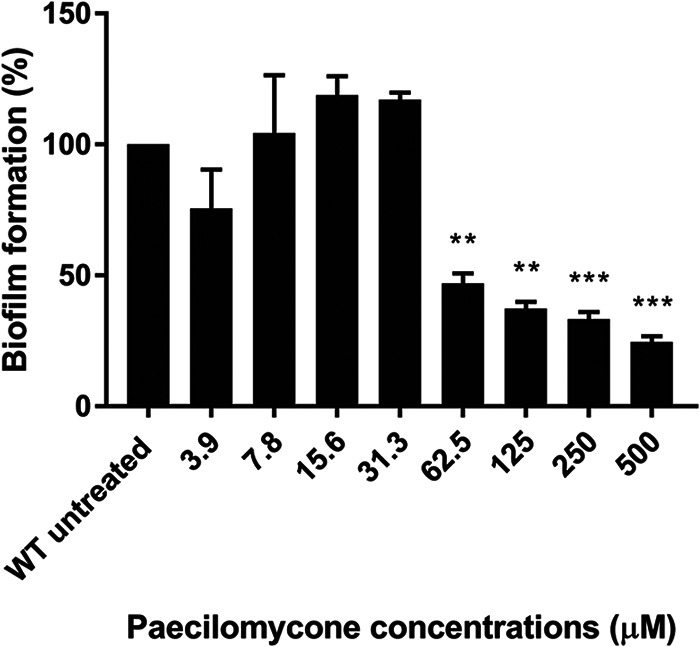
Inhibition of biofilm formation in the P. aeruginosa PAO1 strain by paecilomycone treatment for 24 h. Biofilm formation was measured by crystal violet staining and normalized to the untreated control. The mean of three experiments in triplicate was plotted, and error bars represent SEM. A one-way analysis of variance (ANOVA), corrected for multiple comparisons using Dunnett’s test, was done to determine statistical significance. Treated samples were compared to untreated controls. **, *P* < 0.005; ***, *P* < 0.001.

QS regulates various downstream processes, including biofilm formation (23). Therefore, we tested if paecilomycone inhibits biofilm formation by staining biomass using crystal violet after treatment with various concentrations of paecilomycone for 24 h. Concentrations of 62.5 μM and higher showed a significant concentration-dependent inhibition of biofilm formation in the P. aeruginosa PAO1 strain (Fig. 4). The highest concentration tested (500 μM) showed an inhibition of 75% compared to the WT control. High concentrations affected growth of the bacteria (Fig. S6), which could contribute to decreased biofilm formation. However, the optical density at 600 nm (OD_600_) was similar at 24 h after treatment with 62.5 μM, a concentration that inhibited biofilm formation of P. aeruginosa 50%. Therefore, we conclude that paecilomycone is able to inhibit biofilm formation via mechanisms that are unrelated to growth.

### Paecilomycone alters the production of various virulence factors.

**FIG 5:**
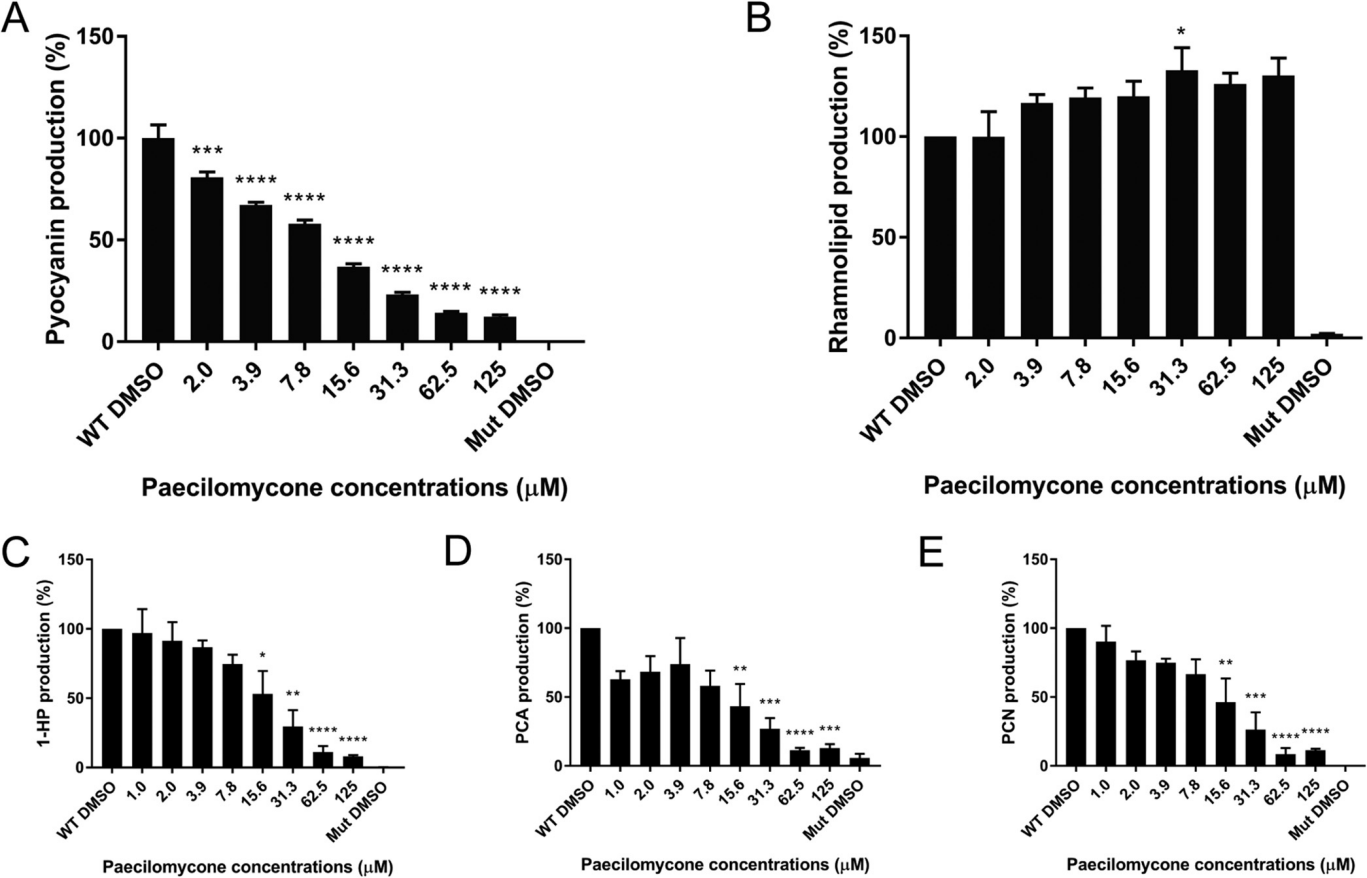
Production of virulence factors by the P. aeruginosa PAO1 strain after paecilomycone treatment. Graphs show the production of (A) pyocyanin, (B) rhamnolipids, (C) 1-hydroxyphenazine (1-HP), (D) phenazine-1-carboxylic acid (PCA), and (E) phenazine-1-carboxamide (PCN) after treatment with paecilomycone for 24 h. A QS mutant (Δ*lasI* Δ*rhlI*) was included as a negative control. Experiments were performed in biological triplicates containing technical triplicates (with the exception of pyocyanin, which was performed once in this setting). Values were normalized to the WT DMSO control, and the mean was plotted; error bars represent SEM. A one-way ANOVA, corrected for multiple comparisons using Dunnett’s test, was done to determine statistical significance. Paecilomycone-treated samples were compared to the WT DMSO control. *, *P* < 0.05; **, *P* < 0.005; ***, *P* < 0.001; ****, *P* < 0.0001.

Besides biofilm formation, QS regulates the synthesis of virulence factors in P. aeruginosa (24–26). Therefore, we tested the effect of paecilomycone treatment on the synthesis of various virulence factors. Paecilomycone induced a strong and significant inhibitory effect on the production of pyocyanin, with a maximum inhibition of 88% and an IC_50_ of 8.5 μM (Fig. 5A). The lowest concentration tested, 2.0 μM, still showed a significant decrease in pyocyanin production. To rule out inadvertent effects on PAO1 due to altered growth, growth kinetics were determined using planktonic cells in King’s A medium, the medium used for analysis of pyocyanin. The initial slopes of the PAO1 bacterial density curves were similar in control and paecilomycone-treated samples, indicating that bacterial growth was similar (Fig. S8). Yet, bacterial density did not reach the same level in samples treated with high paecilomycone concentrations (from 31.3 μM onwards). However, the difference in bacterial densities after 24 h of treatment was relatively small and could not explain the large decrease in pyocyanin production in response to paecilomycone.

Interestingly, rhamnolipid production in PAO1 cells in response to paecilomycone was increased (Fig. 5B). A modest but significant increase (up to 33%) was observed following treatment with 31 μM paecilomycone.

Because the synthesis of pyocyanin showed a strong inhibition, we also measured the effect of paecilomycone on other phenazines produced by P. aeruginosa: 1-hydroxyphenazine (1-HP), phenazine-1-carboxylic acid (PCA), and phenazine-1-carboxamide (PCN) (24). The synthesis of these phenazines showed a significant inhibition of all of them after paecilomycone treatment with a concentration of 15.6 μM or higher (Fig. 5C to E). Maximum inhibition was reached after treatment with 62.5 to 125 μM paecilomycone, with inhibition up to 92% (1-HP), 89% (PCA), and 91% (PCN) compared to the untreated control. This shows that paecilomycone inhibited the production of phenazines, but not rhamnolipids.

### Paecilomycone inhibits production of PQS pathway metabolites.

**FIG 6:**
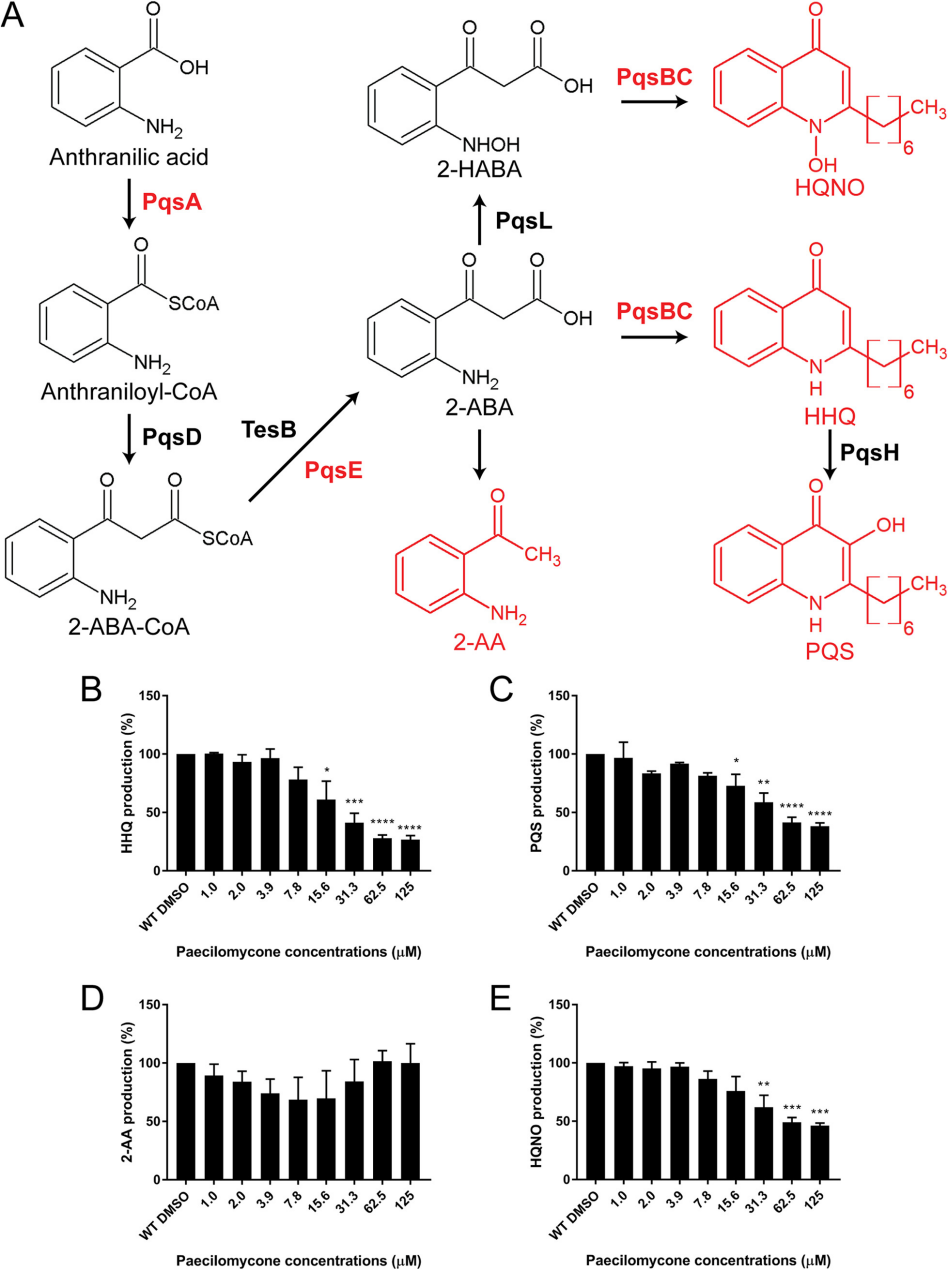
Inhibition of various metabolites in PQS synthesis after paecilomycone treatment. (A) The PQS synthesis pathway starts from anthranilic acid, which is converted by enzymes from the *pqsABCDE* operon to produce HHQ, which is then hydroxylated by PqsH to form PQS. 2-ABA can also spontaneously decarboxylate into 2-AA or be converted into HQNO by PqsL and subsequently the PqsBC complex. Enzymes in red indicate mutants used in Fig. 7. Graphs show the production of the metabolites indicated in red: (B) HHQ, (C) PQS, (D) 2-AA, and (E) HQNO. Experiments were done three times in triplicate, values were normalized to DMSO treated control, and the mean of the experiments is plotted, with error bars representing the SEM. A one-way ANOVA, corrected for multiple comparisons using Dunnett’s test, was done to determine statistical significance. Treated samples were compared to the DMSO control. *, *P* < 0.05; **, *P* < 0.005; ***, *P* < 0.001; ****, *P* < 0.0001.

Since the strongest inhibition of QS was measured in the PQS pathway (Fig. 3) and a strong inhibition was observed in the synthesis of phenazines (Fig. 5), we tested the effect of paecilomycone treatment on the production of various metabolites in PQS synthesis (Fig. 6A). In short, PQS synthesis starts with PqsA, which converts anthranilic acid to anthraniloyl-coenzyme A (CoA) (27–29). After condensation by PqsD, PqsE hydrolyzes 2-aminobenzoylacetyl-CoA (2-ABA-CoA) into 2-aminobenzoylacetyl (2-ABA) (30–32). This hydrolysis can be taken over by TesB thioesterase (32). 2-ABA is condensed to HHQ by the PqsBC complex, which is hydroxylated by PqsH to form PQS (30, 33). In addition, 2-ABA can spontaneously decarboxylate to 2′-aminoacetophenone (2-AA) or form 2-heptyl-4-quinolinol 1-oxide (HQNO) by PqsL and the PqsBC complex (11, 30, 34). To test the effect of paecilomycone on PQS synthesis, we tested the production of 2-AA, HQNO, HHQ, and PQS.

After treatment for 24 h, we measured a significant concentration-dependent inhibition of HHQ and PQS using paecilomycone concentrations of 15.6 μM and higher (Fig. 6B and C). The strongest inhibition was measured after treatment with 125 μM paecilomycone, with inhibition up to 73% and 62% for HHQ and PQS, respectively. Besides HHQ and PQS production, paecilomycone also significantly inhibited HQNO production up to 54% compared to the control (Fig. 6D). Interestingly, the production of 2-AA was not significantly affected after paecilomycone treatment (Fig. 6E). This suggests that paecilomycone treatment did not inhibit the complete PQS synthesis, but only specific enzymes like the PqsBC complex.

To exclude the possibility that paecilomycone C inhibited phenazine production and the PQS pathway, we compared the activity of paecilomycone with that of the fraction without paecilomycone C. We observed no differences, indicating that paecilomycone C is dispensable for inhibition of phenazine production (Fig. S9).

### Paecilomycone is a potential inhibitor of the PqsBC complex.

Since various metabolites of the PQS pathway were inhibited, except for 2-AA, we expected paecilomycone to target the PqsBC complex (Fig. 6A). PqsBC inhibitors have been reported before and show similar patterns to paecilomycone treatment (35–37). To test involvement of the *pqs* genes in the PQS synthesis pathway under our conditions (King’s A medium), we analyzed metabolite expression in PAO1 mutants, which lack genes encoding PQS synthesis enzymes (Δ*pqsA*, Δ*pqsE*, Δ*pqsBC*). We expected the Δ*pqsBC* mutant to show a similar pattern to paecilomycone treatment: i.e., inhibition of HQNO, HHQ, PQS, pyocyanin, and PCA production while not affecting 2-AA.

**FIG 7:**
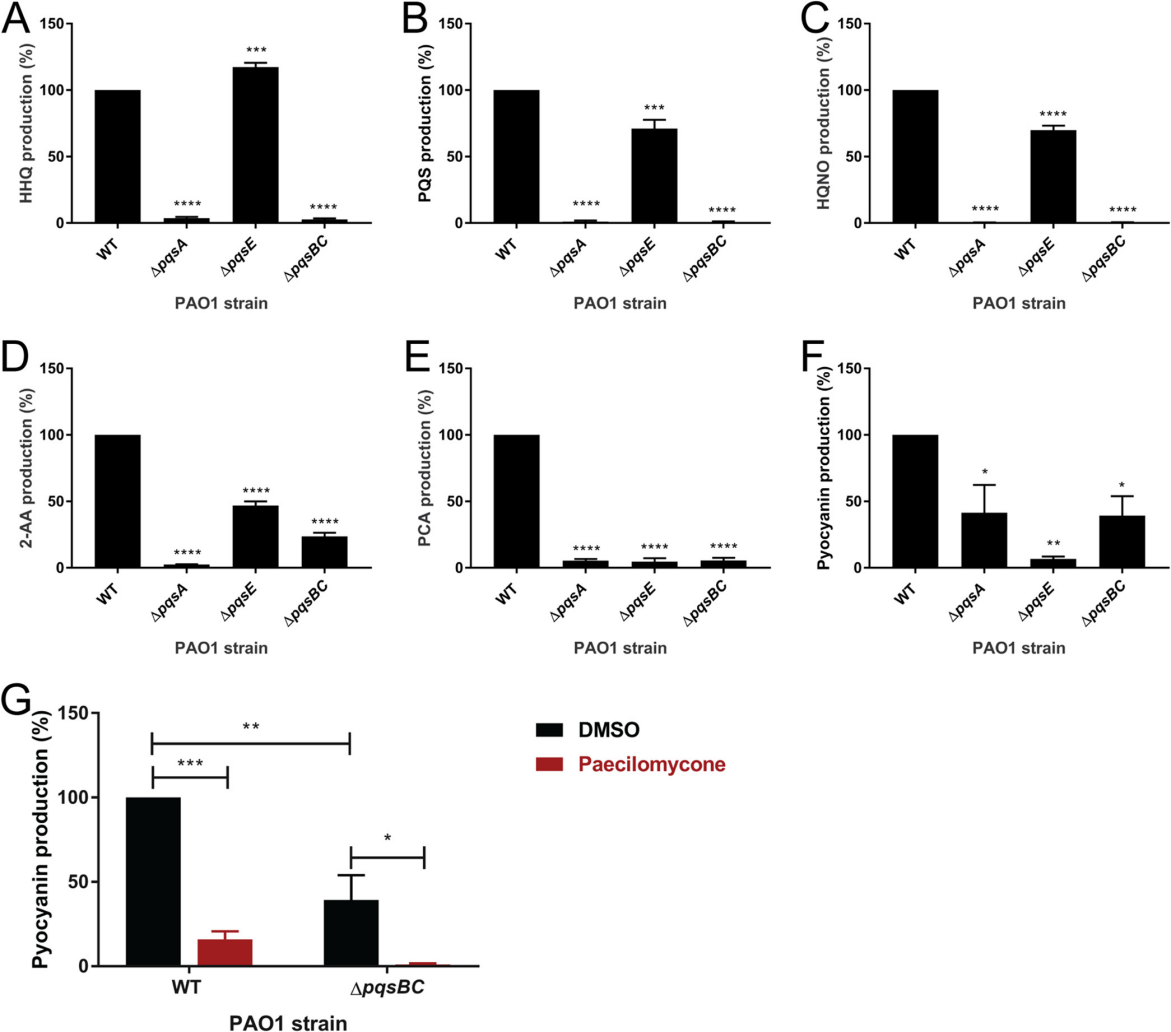
Effect of mutations in PQS synthesis enzymes on the production of various HAQs, phenazines, and 2-AA in King’s A medium. The P. aeruginosa PAO1 strain and various mutants (Δ*pqsA*, Δ*pqsE*, and Δ*pqsBC*) were grown in King’s A medium for 24 h, and the production of (A) HHQ, (B) PQS, (C) HQNO, (D) 2-AA, (E) PCA, and (F) pyocyanin was measured and normalized to the WT control. (G) The WT and Δ*pqsBC* mutants were treated with paecilomycone, and pyocyanin levels were measured. Experiments were done three times in triplicates; the mean of the normalized values is plotted, with error bars representing the SEM. A one-way ANOVA, corrected for multiple comparisons using Dunnett’s test, was done to determine statistical significance. Mutants were compared to the WT control. *, *P* < 0.05; **, *P* < 0.005; ***, *P* < 0.001; ****, *P* < 0.0001.

Indeed, we measured a strong inhibition of both HHQ and PQS in Δ*pqsA* and Δ*pqsBC* strains (Fig. 7A and B). The Δ*pqsE* mutant showed only a slight decrease in PQS and even an increase in HHQ (Fig. 7A and B), which may be due to TesB, which hydrolyzes 2-ABA-CoA to 2-ABA in the absence of PqsE (32) (Fig. 6A). HQNO production was significantly inhibited in all mutant strains, with complete inhibition in both *pqsA* and *pqsBC* mutants (Fig. 7C). In contrast to paecilomycone treatment, which did not affect 2-AA, the *pqsA* mutant showed almost a 100% decrease in 2-AA production, which was expected because PqsA mediates the first step in PQS synthesis (Fig. 7D). Surprisingly, the *pqsBC* mutant still showed a strong decrease in 2-AA production. This may be due to inhibition of the positive feedback of HHQ and PQS on the *pqsABCDE* operon upon complete inhibition of HHQ and PQS production (28).

PqsE regulates the synthesis of pyocyanin. Consistent with this notion, production of pyocyanin and PCA was abolished in *pqsE* mutant. Production of PCA and pyocyanin was also abolished or reduced in *pqsA* and *pqsBC* mutants (Fig. 7E and F). This may be due to positive feedback, leading to reduced expression of PqsE in *pqsA* and *pqsBC* mutants. Together, these data suggest that paecilomycone treatment did not completely inhibit the PQS pathway (cf. Δ*pqsA*) or specifically target phenazine synthesis (cf. Δ*pqsE*) but rather that paecilomycone inhibited aspects of PQS synthesis (cf. Δ*pqsBC*).

Interestingly, while the *pqsBC* mutant already showed decreased levels of pyocyanin, treatment with paecilomycone strengthened this inhibition significantly to almost 100% (Fig. 7G). This suggests that paecilomycone might have alternative targets or targeted a process related to the enzymatic activity of the PqsBC complex instead of directly targeting the PqsBC complex, which caused an even stronger inhibition of pyocyanin in the absence of the PqsBC complex.

### Toxicity of paecilomycone.

**FIG 8:**
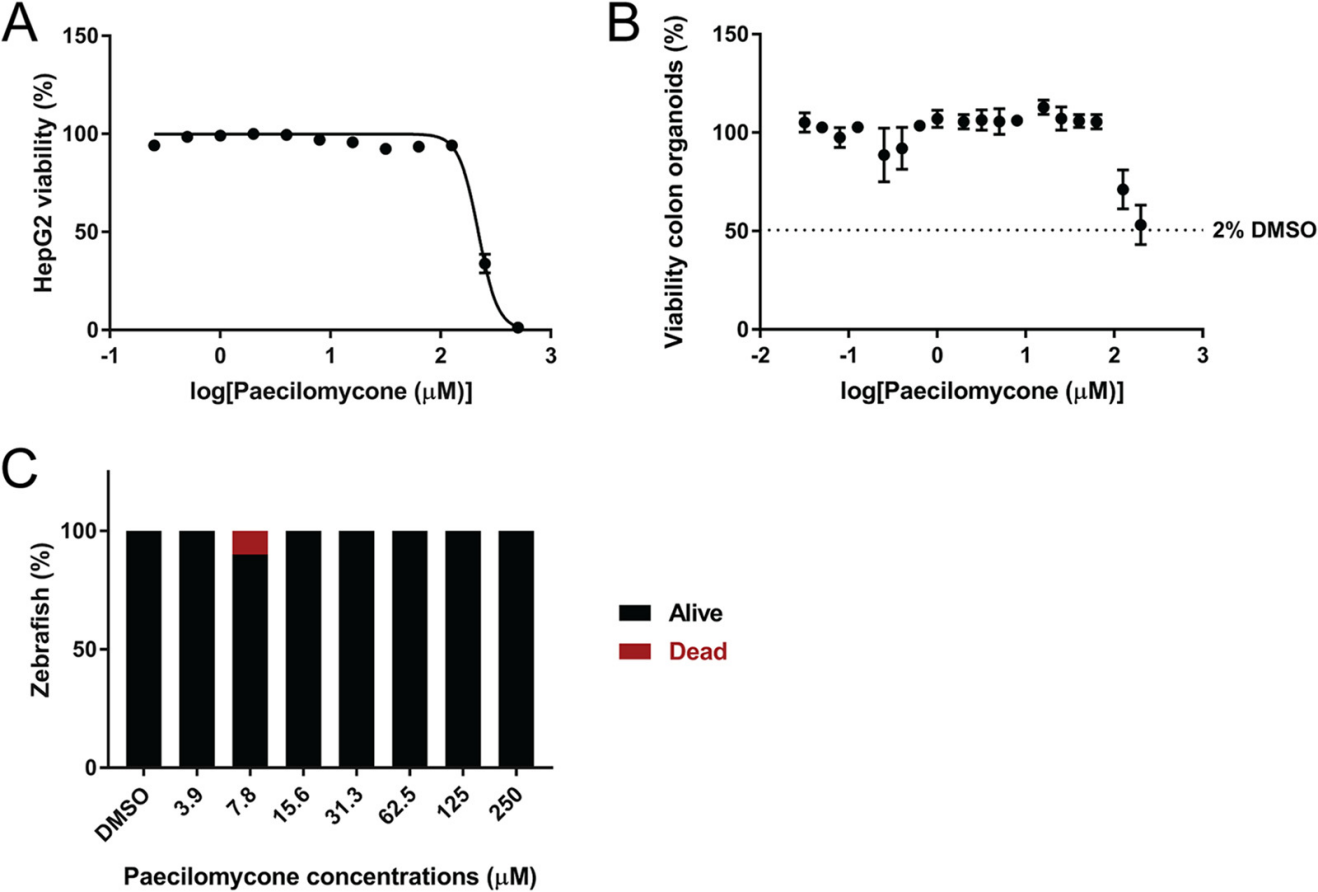
Toxicity of paecilomycone treatment using various toxicity models. (A) Viability of HepG2 cells treated with paecilomycone for 24 h. Viability was measured using the resazurin assay. (B) Viability of colon organoids treated with paecilomycone for 5 days. Viability was measured by measuring ATP levels by a Cell Titer Glo 3D assay. (C) Viability of zebrafish embryos that were treated after 48 h for 24 h. Experiments were done in triplicate and error bars indicate SEM.

To test if paecilomycone has clinical potential, we determined the cytotoxicity of paecilomycone on various viability models. First, we used human liver-derived HepG2 cells. Viability of HepG2 cells was assessed after paecilomycone treatment for 24 h. Up to a concentration of 125 μM paecilomycone, there was no difference in the viability of HepG2 cells compared to untreated control cells (Fig. 8A). Paecilomycone was toxic to HepG2 cells only at high concentrations, with an IC_50_ of 219 μM.

Second, we tested the cytotoxic effect of paecilomycone on more complex systems, human colon organoids. Viability of the organoids was measured after paecilomycone treatment for 5 days. High concentrations (200 μM and 120 μM) showed reduced viability compared to 1% dimethyl sulfoxide (DMSO) (Fig. 8B). However, at these high concentrations, the final concentration of paecilomycone solvent was higher than 1%, up to 2% DMSO, which by itself already showed a 50% reduction in viability. It is therefore highly likely that the apparent effect of 120 μM and 200 μM paecilomycone on organoid viability was actually caused by high concentrations of DMSO.

Third, we tested the cytotoxic effect of paecilomycone on a whole organism *in vivo*, 2-day-old zebrafish embryos. The viability of the embryos was measured after paecilomycone treatment for 24 h. Each concentration was tested on ~10 embryos. No toxic effect of paecilomycone was apparent (Fig. 8C).

In conclusion, paecilomycone did not affect eukaryotic cells at concentrations that inhibited QS in Gram-negative bacteria. Only at high paecilomycone concentrations was an effect detected on HepG2 cells, whereas no effect was specifically attributable to paecilomycone treatment of more complex systems, like organoids and zebrafish embryos.

## DISCUSSION

In this study, we describe the identification of paecilomycone as a novel QS inhibitor. Paecilomycone showed inhibition of QS in Gram-negative bacterial strains of C. violaceum and P. aeruginosa PAO1. Paecilomycone showed the strongest inhibitory effect on the PQS pathway, and it strongly inhibited the production of HHQ and PQS. In addition, paecilomycone inhibited biofilm formation and the production of phenazines, including pyocyanin. Paecilomycone might have promising clinical value because cytotoxicity was very low.

Paecilomycone treatment showed strong inhibition of QS in the *pqsA*-GFP reporter strain (Fig. 3), production of related virulence factors (Fig. 5), and production of HHQ and PQS (Fig. 6). Since paecilomycone did not interfere with the production of 2-AA (Fig. 6), we suggest that paecilomycone affected the activity of the PqsBC complex. Only a limited number of PqsBC inhibitors have been identified so far. Previously described PqsBC inhibitors show similar profiles to paecilomycone: inhibition of HHQ and PQS, while 2-AA is unaffected or even upregulated (35–37). Only dual PqsBC and PqsR inhibitors showed inhibition of 2-AA, something that is comparable to our Δ*pqsBC* mutant strain (35–37). Unfortunately, these studies do not describe the effect of the PqsBC inhibitors on expression of virulence traits in P. aeruginosa, except for a small decrease in pyocyanin production, whereas we observed a strong inhibition after paecilomycone treatment (35). Based on our data, the PqsBC complex is the most likely target of paecilomycone treatment.

It was unexpected that the paecilomycone targets the PQS system in P. aeruginosa after we identified its QS inhibitory effect in C. violaceum. Although both bacteria contain a LuxI/R-type QS, the PQS system is unique for P. aeruginosa (9–11, 21). More specifically, the PqsBC complex is a FabH-like enzyme that catalyzes the condensation of an octanoyl-CoA with 2-ABA (38, 39). This complex does not show similarity to the CviI synthase in C. violaceum. Therefore, interaction of paecilomycone with a LuxI/R-type system of P. aeruginosa would be expected. However, there are several reasons we believe paecilomycone has a different mechanism of action in C. violaceum than in P. aeruginosa.

In P. aeruginosa, paecilomycone induced *lasB*-GFP reporter inhibition, whereas *rhlA-*GFP was not affected. The Las regulatory system is believed to be on top of the QS hierarchy, positively regulating the Rhl and PQS system (10). Therefore, inhibition of Las might also lead to inhibition of PQS. However, this inhibition would lead to complete inhibition of the entire *pqsABCDE* operon and thus to inhibition of 2-AA, which we did not observe. Moreover, conditions with low phosphate concentrations (like King’s A medium, used in this study for the pyocyanin assay and phenazine and HAQ assay) might bypass Las, resulting in direct activation of Rhl and PQS. Note that because of this bypass, LasR inhibitors are not effective in low phosphate conditions (40). Therefore, inhibition of the *lasB-*GFP reporter might be due to positive regulation of *lasR* by PqsR (41), instead of direct inhibition by paecilomycone.

Inhibition of pyocyanin production was also detected after activation of the Rhl pathway, because RhlR inhibits the *pqsABCDE* operon (42). Therefore, another explanation is that paecilomycone acts as an RhlR agonist. However, similar to inhibition of the Las system, RhlR activation would also lead to inhibition of 2-AA. Therefore, unless the production of 2-AA can be bypassed somehow, we favor the explanation that paecilomycone affects the activity of the PqsBC complex.

PqsBC complex might be a direct target of paecilomycone. PqsE regulates the production of phenazines via RhlR (43–49). A mutant lacking PqsE did not produce PCA or pyocyanin, underlining the importance of PqsE for production of these phenazines. The observed inhibition of PCA and pyocyanin production in the Δ*pqsA* and Δ*pqsBC* mutants may be explained by inhibition of the *pqsABCDE* operon via inhibition of a feed-forward loop, which eventually results in reduced expression of PqsE. However, paecilomycone treatment did not inhibit 2-AA production; therefore, we do not expect a strong inhibition of the entire *pqsABCDE* operon by paecilomycone treatment. Moreover, paecilomycone still had a significant inhibitory effect on pyocyanin production in the Δ*pqsBC* strain (Fig. 7G), suggesting that the PqsBC complex cannot be the only target of paecilomycone. Because of the strong inhibition of phenazines and inhibition of pyocyanin in the *pqsBC* mutant, we believe that paecilomycone did not target only the PqsBC complex. Instead, we suggest that paecilomycone affects QS by directly targeting the PqsBC complex and alternative targets, or paecilomycone alters processes that influence the enzymatic activity of the PqsBC complex. Further research will be needed to study if paecilomycone indeed targets the PqsBC complex and to elucidate the exact mechanism of action of paecilomycone.

Paecilomycone consisted of three different paecilomycones. Analytical HPLC analysis showed that the concentration of paecilomycone A is highest (Fig. 1). To validate that paecilomycone A is responsible for the measured activity, we tested the purified fraction without paecilomycone C (see Fig. S5 in the supplemental material) in various assays. Both in C. violaceum (Fig. 2) and in the inhibition of phenazines and HAQs (Fig. S9), we measured similar activities between paecilomycone and the fraction lacking paecilomycone C, suggesting that paecilomycone C was not required for the effect of paecilomycone in QS inhibition. Interestingly, although funalenone shows high resemblance to paecilomycone A, funalenone did not show inhibition of QS in C. violaceum (Fig. 2) or P. aeruginosa (Fig. S7). This suggests that the position of the methoxy group, which is the main difference between paecilomycone and funalenone, is important in the QS inhibitor activity.

Inhibition of HAQ and phenazine production is an interesting method to fight the virulence of P. aeruginosa. Phenazines are among the major virulence factors produced by P. aeruginosa, and they play an important role in acute and chronic lung infections through various mechanisms (50–54). Phenazines have been shown to be toxic in various model organisms, including Caenorhabditis elegans (55), Drosophila melanogaster (56), mice (57), and epithelial lung cells (53, 54). In addition, in humans, phenazine production has been measured in sputum samples in concentrations that are able to inhibit ciliary beating, which is important for the clearance of bacteria (58). Moreover, phenazines are also involved in biofilm formation (59) and in the acquisition of iron (60). In general, phenazines are important for the survival of bacteria and for damaging the host. Therefore, inhibition of phenazines by paecilomycone holds potential as an interesting therapeutic lead, by reducing toxicity and subsequently morbidity and mortality in patients.

Whereas the effect of paecilomycone on phenazine synthesis was strong, with an IC_50_ of 8.5 μM for pyocyanin synthesis, this inhibitory effect was less strong on biofilm formation. The inhibition of biofilm formation after treatment of paecilomycone at concentrations higher than 125 μM might be explained by effects on growth. However, a concentration of 62.5 μM still inhibited biofilm formation 50%, without affecting growth. Next to QS, there are various pathways that control biofilm formation, including c-di-GMP signaling and the Gac/Rsm cascade (23). This might explain the difference in effective concentrations for inhibition of biofilm formation and phenazine production.

Because of its potential to inhibit QS and downstream targets like phenazine production and biofilm formation, we believe paecilomycone is an interesting lead for further clinical research. We showed that concentrations that inhibit virulence factor production and biofilm formation were not toxic to cell cultures. Moreover, we did not measure toxic effects in more complex systems up to the highest concentrations tested (200 to 250 μM). The differences in toxicity between HepG2 cells on the one hand and organoids and zebrafish on the other might be attributed to the fact that complex systems have higher capacity to negotiate and/or abolish the homeostasis and toxic effects of drug compounds. Taken together, our results suggest that paecilomycone is an interesting lead for further research as a potential agent to fight P. aeruginosa infections.

## MATERIALS AND METHODS

### Bacterial strains and growth conditions.

The bacterial strains used in this study (see Table S1 in the supplemental material) were stored at −80°C in 20% glycerol stock solutions. C. violaceum was plated on tryptic soy agar (TSA) and grown in tryptic soy broth (TSB) at 27°C. PAO1 strains were grown on Luria agar (LA) plates at 37°C and grown in medium specific for the assay. Escherichia coli RHO3 strains were used for conjugation, and medium was supplemented with 400 μg/mL 2,6-diaminopimelic acid (Sigma-Aldrich, Merck Life Science, Amsterdam, the Netherlands) to support growth.

Mutants were generated using allelic exchange following the method described before (61). For the generation of mutants, we inserted upstream (UP.Fw and UP.Rv primers used) and downstream (DN.Fw and DN.Rv primers used) regions of the gene of interest in pEX18Gm vector (a gift from Joe Harrison, University of Calgary) using Gibson assembly restriction cloning with the restriction enzymes SacI and SphI (Tables S2 and S3). After Gibson assembly, the vector containing the regions flanking the gene of interest was transformed into RHO3 E. coli donor strains before conjugation with WT PAO1 cells. Mutant cells were identified by colony PCR (seq.Fw and seq.Rv primers used) and confirmed by sequencing (performed by Macrogen Europe BV).

### Chemical analysis.

**(i) Purification of paecilomycone.** Paecilomycone was purified using the same method as described before, with minor changes (20). In brief, the fungus Aspergillus allahabadii was grown on a malt extract agar (MEA) plate at 25°C. After 7 days, cubes of 5 by 5 mm were cut out, and 2 cubes were inoculated per 100-mL bottle containing 50 mL of potato dextrose broth (PDB). The liquid culture was inoculated at 25°C with 100-rpm orbital shaking. After 7 days, the liquid medium was filter sterilized using a 0.22-μm-pore Millipore filter (Merck, Amsterdam, The Netherlands). The sterile supernatant was extracted using 3× 1/3 vol of ethyl acetate and evaporated to dryness using a rotary evaporator with a water bath at 40°C. The dried pellet was dissolved in DMSO.

The extract was fractionated using a preparative high-performance liquid chromatography (HPLC) system consisting of a Shimadzu CBM-20A controller, a Shimadzu LC-20AP pump, and a Shimadzu FRC-10A fraction collector with a C_18_ reversed-phase Reprosil column (10 μm, 120 Å, 250 by 22 mm) and a Shimadzu SPD-20A UV-detector set at 214 nm and 254 nm. The mobile phase consisted of 100% Milli-Q (MQ) with 0.1% trifluoroacetic acid (buffer A) and 100% acetonitrile with 0.1% trifluoroacetic acid (buffer B). The protocol consisted of 5% buffer B for 5 min, followed by a linear gradient to 95% buffer B for 40 min, then 5 min of 95% buffer B, before returning to 5% buffer B for another 5 min, with a constant flow rate of 12.5 mL/min. The active fraction with a retention time of 31 min was collected and dried overnight using a SpeedVac.

The next day, the dried pellet was dissolved in DMSO and fractionated again using the same preparative HPLC system, but with different buffers. Buffer A consisted of 95:5 MQ-acetonitrile plus 10 mM NH_4_OAc. Buffer B consisted of 80:20 acetonitrile-MQ plus 10 mM NH_4_OAc. The protocol consisted of 0% buffer B for 5 min, followed by a linear gradient to 100% buffer B for 40 min, then 5 min of 100% buffer B, before returning to 0% buffer B for another 5 min, with a constant flow rate of 12.5 mL/min. Paecilomycone had a retention time of 23 min and was collected and dried using a SpeedVac.

Paecilomycone was dissolved in a stock concentration of 40 mM in DMSO and stored at −20°C. Purity was checked using a Shimadzu LC-2030C 3D Plus analytical HPLC system with photodiode array (PDA) detection (190 to 800 nm) with a Dr. Maisch Reprosil-PUR 120 C_18_ AQ column (3 μm, 120 Å, 4.6 by 100 mm). The mobile phase consisted of 100% MQ with 0.1% trifluoroacetic acid (buffer A) and 100% acetonitrile with 0.1% trifluoroacetic acid (buffer B). The protocol consisted of a linear gradient from 5% to 95% buffer B for 10 min, followed by 2.5 min of 95% buffer B, before returning to 5% buffer B, with a constant flow rate of 1 mL/min.

**(ii) Identification of paecilomycone.** The UV-Vis spectrum of paecilomycone was obtained using analytical HPLC methods mentioned above. The compound was further analyzed by measuring the mass using the same LC system with a Shimadzu LCMS-2020 mass spectrometer. For LC-MS analysis, the mobile phase consisted of 100% MQ with 0.05% formic acid (buffer A) and 100% acetonitrile with 0.05% formic acid (buffer B). The protocol consisted of a linear gradient from 5% to 95% buffer B for 10 min, followed by 2.5 min of 95% buffer B, before returning to 5% buffer B, with a constant flow rate of 0.5 mL/min. The mass spectrometry settings were as follows: nebulizing gas flow of 1.5 L/min, drying gas flow of 15 L/min, desolvation line temperature of 250°C, capillary voltage of 4,500 V for positive mode and 3,500 V for negative mode, mass range of 100 to 1,000 *m/z*, and scan speed of 5,000 *u*/s. Paecilomycone A had a retention time of 12.7 min, paecilomycone B had a retention time of 9.8 min, and paecilomycone C had a retention time of 8.9 min.

This was followed by a more accurate high-resolution mass spectrometry using an LCT instrument (Micromass, Ltd., Manchester, United Kingdom). The instrument was calibrated using sodium formate, followed by direct injection of the sample with the following settings: cone gas flow of 50 L/h, desolvation gas flow of 250 L/h, desolvation temperature of 120°C, source temperature of 80°C, capillary voltage of 3,000 V, microchannel plate (MCP) detector at 2,450 V, reflectron at 1,786 V, time of flight (TOF) tube at 4,567 V, mass range of 150 to 750 *m/z*, and scan speed of 600 *u*/s.

For nuclear magnetic resonance (NMR) analysis, paecilomycone was dissolved in DMSO-*d_6_*. The NMR measurements (^1^H, ^13^C heteronuclear single-quantum correlation [HSQC] and heteronuclear multiple-bond correlation spectroscopy [HMBC]) were performed on a Bruker 600-MHz device.

### Quorum sensing inhibition in C. violaceum.

To measure the effect on QS inhibition in C. violaceum, we used a protocol based on previous work, with minor changes (62). In brief, C. violaceum was plated on tryptic soy agar (TSA) and grown in TSB overnight at 27°C. The next morning, bacteria were diluted and grown until an optical density at 600 nm (OD_600_) of 0.5 to 0.7. Then, bacteria were diluted 1,000-fold and added to a 96-well plate containing paecilomycone in serial dilutions up to a volume of 100 μL (range, 31 nM to 1 mM). High concentrations of DMSO are toxic to the bacteria, and therefore the maximum concentration of DMSO was limited to 2.5%. Plates were incubated for 20 h at 27°C while shaking at 180 rpm.

To measure violacein production, the plate was centrifuged for 10 min at 3,000 rpm to pellet the violacein. Subsequently, the supernatant was discarded and the violacein pellet was dissolved in 200 μL of 96% ethanol. The plate was centrifuged for 10 min at 3,000 rpm to avoid interference of cell turbidity in absorbance measurements. Half of the supernatant was then transferred to a new 96-well plate, and violacein was quantified by measuring the OD_562_ on the ASYS Expert Plus microplate reader (Biochrom, Ltd., Cambridge, United Kingdom).

To measure the effect of paecilomycone on viability of the bacteria, resazurin staining was used. In parallel with the measurements of violacein production, another plate with C. violaceum and paecilomycone dilutions was prepared and incubated overnight. The next morning, the plate was centrifuged for 10 min at 3,000 rpm to pellet the bacterial cells. The supernatant was aspirated, and 0.1 mM resazurin (in phosphate-buffered saline [PBS]) solution was added. Plates were incubated for another 45 min at 27°C before fluorescence was measured on a PHERAstar microplate reader (BMG Labtech) using 540-nm excitation and 590-nm emission wavelengths.

### Quorum sensing inhibition in P. aeruginosa.

The experiments were performed as previously described (63). In brief, P. aeruginosa PAO1 reporter lines were grown overnight in AB minimal medium supplemented with 0.5% glucose and 0.5% Casamino Acids. Cultures were diluted until an OD_450_ of 0.1 to 0.2 before being added to a 96-well plate containing serial dilutions of paecilomycone up to a volume of 200 μL (range, 4 nM to 500 μM). The GFP fluorescence (excitation, 485 nm; emission, 535 nm) and absorbance (600 nm) were measured every 15 min for 15 h at 34°C on a CLARIOstar microplate reader (BMG Labtech). IC_50_ values were calculated using Prism software, plotting the maximum slope of GFP/OD_600_.

### Growth curves.

P. aeruginosa was grown overnight in desired medium. The next day, bacteria were diluted in PBS until an OD_600_ of 0.1 to 0.2. Diluted bacteria were added to a honeycomb microplate containing the desired medium with paecilomycone up to a volume of 300 μL (1:1 [vol/vol]). The absorbance (600 nm) was measured every 15 min for 24 h at 34°C on a Bioscreen C (Growth Curves Ab, Ltd.).

### Biofilm assay.

Bacteria were grown overnight in AB minimal medium supplemented with 0.5% glucose and 0.5% Casamino Acids before being diluted 1,000-fold. Diluted bacterial cells were added to a 96-well plate containing paecilomycone in serial dilutions (range, 3.9 μM to 500 μM) in triplicates to a final volume of 200 μL. Plates were sealed with Breathe-Easy sealing membrane (Sigma-Aldrich, Merck Life Science, Amsterdam, The Netherlands) to prevent evaporation and incubated at 37°C under static conditions for 24 h. The next day, the medium was removed and the wells were rinsed with PBS. Biomass was stained with 0.1% (wt/vol) crystal violet solution for 5 min. Crystal violet was discarded, and excess crystal violet was removed by rinsing with water. Plates were dried, and bound crystal violet was dissolved in 33% (vol/vol) acetic acid and quantified at 562 nm using an ASYS Expert Plus microplate reader (Biochrom, Ltd., Cambridge, United Kingdom).

### Virulence factor assays.

**(i) Pyocyanin assay.** The pyocyanin assay was performed as previously described, with minor modifications (64). Bacteria were grown overnight in King’s A medium (2% [wt/vol] protease peptone, 1% [wt/vol] potassium sulfate, 0.164% [wt/vol] magnesium chloride, 1% [vol/vol] glycerol in MQ), before diluting them 100-fold. The assay was performed in bacterial tubes containing 1 mL of diluted bacterial culture in combination with paecilomycone at desired concentrations (range, 2 μM to 125 μM). A Δ*lasI* Δ*rhlI* QS mutant strain was included as a negative control. The tubes were incubated at 37°C on an orbital shaker set at 180 rpm. After 24 h, bacterial cells were pelleted by centrifugation at 4,000 rpm for 10 min and 900 μL supernatant was extracted with a similar volume of chloroform. Eight hundred microliters of chloroform was then added to 700 μL of 0.2 M HCl, and the samples were mixed well. The HCl phase was then measured at 520 nm to measure relative pyocyanin concentrations.

**(ii) Rhamnolipid assay.** The rhamnolipid assay was performed as previously described, with minor modifications (65). Bacteria were grown overnight in AB minimal medium supplemented with 0.5% glucose and 0.5% Casamino Acids before being diluted 100-fold. The assay was performed in bacterial tubes containing 1 mL of diluted bacterial culture in combination with paecilomycone at the desired concentration (range, 2 μM to 125 μM). A Δ*lasI* Δ*rhlI* QS mutant strain was included as a negative control. The tubes were incubated at 37°C on an orbital shaker set at 180 rpm. After 24 h, bacterial cells were pelleted, 900 μL of supernatant was added to diethyl ether (1:1 [vol/vol]), and the tubes were shaken vigorously. The diethyl ether layer was then transferred to a fresh tube and dried at room temperature. One hundred microliters was added to dissolve the dried pellet before addition of 800 μL of 12.9 mM orcinol (Sigma-Aldrich, Merck Life Science, Amsterdam, the Netherlands) in 70% (vol/vol) H_2_SO_4_. The reaction mixture was maintained at 80°C for 30 min before measuring the absorbance at 495 nm.

**(iii) Phenazine and HAQ assay.** The phenazine and HAQ assay was based on the pyocyanin assay, with minor modifications. Bacteria were grown in King’s A medium before 100-fold dilution. The assay was performed in bacterial tubes containing 3 mL of diluted bacterial culture in combination with paecilomycone at the desired concentrations (range, 1 μM to 125 μM). A Δ*lasI* Δ*rhlI* QS mutant strain was included as negative control. The tubes were incubated at 37°C with 180-rpm orbital shaking. After 24 h, the bacterial cells were pelleted and the supernatant was extracted with 2× 1 mL chloroform. Chloroform was dried overnight, and the pellet was dissolved in DMSO. Extracts were run using previously described analytical HPLC methods, with a slightly altered gradient: buffer B gradient was used from 5% to 95% for 20 min, followed by 2.5 min of 95% buffer B, before returning to 5% buffer B, with a constant flow rate of 1 mL/min. Compounds were quantified by using calibration curves made by serial dilutions of standard commercial phenazines and HAQs. If needed, extra verification of the compounds was performed using previously described LC-MS methods.

### Toxicity assays.

**(i) HepG2 toxicity assay.** HepG2 cells were seeded into 96-well plates and grown in Dulbecco’s modified Eagle’s medium (DMEM) low-glucose medium (Thermo Fisher Scientific; 10567014) supplemented with 10% fetal bovine serum (FBS). Cells were grown until a confluence of ~70 to 80% before addition of paecilomycone (range, 244 nM to 500 μM) in triplicates, with a final DMSO concentration of 1% DMSO. Treated cells were incubated at 37°C with 5% CO_2_ for 24 h. To measure the viability of the cells, 0.1 mM resazurin (Sigma-Aldrich) solution was added, and cells were incubated for another 3 h. Fluorescence intensity was measured on a PHERAstar microplate reader (BMG Labtech), using an excitation wavelength of 540 nm and emission wavelength of 590 nm.

**(ii) Organoid toxicity assay.** Human colon tissue was obtained from the UMC Utrecht with patient informed consent. The patient was a male diagnosed with small colon adenocarcinoma. After resection, a sample from nontransformed, normal mucosa was taken for this study. This study was approved by the UMC Utrecht (Utrecht, the Netherlands) ethics committee and was in accordance with the Declaration of Helsinki and according to Dutch law. This study is compliant with all relevant ethical regulations regarding research involving human participants. All organoid experiments were performed in the lab of Hans Clevers.

Human colon organoids were maintained in human colon expansion medium as previously described (66). The toxicity assay was largely performed as described elsewhere (67). In brief, 2 days after the previous split, organoids were dissociated from the Cultrex basement membrane extract (BME) (R&D Biosystems, Bio-Techne; 3533-001-02) using dispase (Thermo Fisher Scientific; 17105-041) for 30 min at 37°C. Then, organoids were washed in advanced DMEM–F-12 (Thermo Fisher Scientific; 12634-010) supplemented with penicillin-streptomycin (Thermo Fisher Scientific, 15140122), HEPES (Thermo Fisher Scientific; 15630080), and GlutaMAX (ThermoFisher Scientific, 35050061) (here called washing medium), filtered through a 70-μm-pore cell strainer (Greiner), and pelleted at 500 × *g* for 5 min. The pellet was resuspended in 1 mL of washing medium, and organoids were counted. Organoids were resuspended at a concentration of 18,750 organoids/mL in 5% cold BME–95% cold human colon expansion medium. Forty microliters of organoid suspension (750 organoids) was dispensed in each well of a 384-well-plate (Corning; 4588) using a multidrop Combi reagent dispenser (Thermo Fisher Scientific; 5840300). Paecilomycone was added immediately after plating of the organoids at concentrations ranging from 10 nM to 200 μM using the Tecan D300e digital dispenser (Tecan). The stock concentration was 10 mM, and hence, the highest concentration contained 2% DMSO. Therefore, a 2% DMSO-only viability control was added. Organoids were incubated in a humidified incubator with 5% CO_2_ at 37°C for 5 days. After 5 days, the ATP levels were measured by a Cell Titer Glo 3D assay (Promega; G9681) following the manufacturer’s instructions. Luminescence was measured using a Spark multimode microplate reader (Tecan) with an integration time of 500 ms. Results were normalized to 1% DMSO control (100% viability) and 1 μM staurosporine (0% viability). Concentrations were measured in triplicates.

**(iii) Zebrafish toxicity assay.** Zebrafish eggs were obtained from Tubingen long fin family crosses. The zebrafish embryos were allowed to develop normally for 48 h. At 48 h postfertilization (hpf), the embryos were divided over 24-well plates, 10 embryos per well in 1 mL of fresh E3 medium. Subsequently, paecilomycone was added in serial dilutions to the wells (range, 3.9 nM to 250 μM). The effects were scored at 72 hpf.

All procedures involving experimental animals were approved by the local animal experiments committee (Koninklijke Nederlandse Akademie van Wetenschappen-Dierexperimentencommissie) and performed according to local guidelines and policies in compliance with national and European law. Adult zebrafish were maintained as previously described (68).

### Commercial compounds used.

The compounds 2′-aminoacetophenone (2-AA), 4-hydroxy-2-heptylquinoline (HHQ), 2-heptyl-3-hydroxy-4(1H)-quinolone (PQS) (Sigma-Aldrich, Merck Life Science, Amsterdam, the Netherlands), 2-heptyl-4-quinolinol 1-oxide (HQNO), 1-phenazinecarboxylic acid (PCA), and phenazine-1-carboxamide (PCN) (Caymen Chemicals) were used to make calibration curves to quantify molecules in bacterial extract. Funalenone (AdipoGen Life Sciences) was used to compare activity with paecilomycone.
