# Supplementary material for: Paecilomycone Inhibits Quorum Sensing in Gram-Negative Bacteria
Source: Microbiol Spectr. 2023 Mar 15;11(2):e05097-22. doi: 10.1128/spectrum.05097-22 (PMC10100902; doi:10.1128/spectrum.05097-22)
Supplement: Supplemental file 1 — Supplemental material. Download spectrum.05097-22-s0001.pdf, PDF file, 6.6 MB [file spectrum.05097-22-s0001.pdf]

Supplementary Table 1: Bacterial strains used in this study

Bacterial strain	***Characteristic***	***Source***
Chromobacterium violaceum	WT, ATCC 12472	Westerdijk Fungal Biodiversity Institute
P. aeruginosa	WT, PAO1	
PAO1-GFP	WT, PAO1 GFP-tagged	Yang *et al.* (2007)(1)
PAO1 lasB-GFP	WT, PAO1, GFP fusion to *lasB* gene	Hentzer *et al.* (2002)(2)
PAO1 rhlA-GFP	WT, PAO1, GFP fusion to *lasB* gene	Fong *et al.* (2017)(3)
PAO1 pqsA-GFP	WT, PAO1, GFP fusion to *pqsA* gene	Fong *et al.* (2017)(3)
PAO1 ΔlasI-ΔrhlI	PAO1, QS mutant	Hentzer *et al.* (2003)(4)
PAO1 ΔpqsA	PAO1, *pqsA* mutant	This study
PAO1 ΔpqsE	PAO1, *pqsE* mutant	This study
PAO1 ΔpqsBC	PAO1, *pqsBC mutant*	This study
E. coli RHO3	Conjugation proficient *E. coli*	Gift from University Medical Center Utrecht

Supplementary Table 2: Primers used in this study

Primer name	Primer sequence
pqsA.UP.Fw	GCCAGTGCCAAGCTTGCATGCATGTAGGTGTCCTCTTC
pqsA.UP.Rv	GGTGTCGGCCAGGAACAGAACCTCGGTCAGG
pqsA.DN.Fw	GTTCTGTTCCTGGCCGACACCCTTT
pqsA.DN.Rv	CATGATTACGAATTCGAGCTGCCTACCTGGCGAATATC
pqsA.seq.Fw	TCCCGTTCCTGACAAAGCAA
pqsA.seq.Rv	CACACATAGGATGGGGGCAG
pqsE.UP.Fw	GCCAGTGCCAAGCTTGCATGTGTCCAAGCGCATGGACTG
pqsE.UP.Rv	GTCTCAGTCCAGCCTCAACATGGCCGG
pqsE.DN.Fw	CCATGTTGAGGCTGGACTGAGACGGGACAT
pqsE.DN.Rv	CATGATTACGAATTCGAGCTAGGTCGAAGCTGAACAGG
pqsE.seq.Fw	GGACGACATCGACCATGTGA
pqsE.seq.Rv	CGTGCGGTACTCCAGACTTT
pqsBC.UP.Fw	GCCAGTGCCAAGCTTGCATGAGGGCTGAGTCCGGGTTAC
pqsBC.UP.Rv	TCACCCAATTGGTTCACCCCCACAGCC
pqsBC.DN.Fw	GGGGGTGAACCAATTGGGTGAGGTGCTGGT
pqsBC.DN.Rv	CATGATTACGAATTCGAGCTGTCTTCGAGACTCTCGCC
pqsBC.seq.Fw	CTGGCCGACACCCTTTATCA
pqsBC.seq.Rv	ATGATTGCTGACCTGGCGTT

Supplementary Table 3: Plasmids generated in this study

Plasmid	***Use***
pEX18Gm::Δ*pqsA*	Plasmid containing *pqsA* deletion construct for use in PAO1
pEX18Gm::Δ*pqsE*	Plasmid containing *pqsE* deletion construct for use in PAO1
pEX18Gm::Δ*pqsBC*	Plasmid containing *pqsBC* deletion construct for use in PAO1

References

1. Yang L, Barken KB, Skindersoe ME, Christensen AB, Givskov M, Tolker-Nielsen T. 2007. Effects of iron on DNA release and biofilm development by Pseudomonas aeruginosa. Microbiology 153:1318–1328.

2. Hentzer M, Riedel K, Rasmussen TB, Heydorn A, Andersen JB, Parsek MR, Rice SA, Eberl L, Molin S, Hoiby N, Kjelleberg S, Givskov M, Høiby N, Parsek MR, Riedel K, Heydorn A, Eberl L, Andersen JB, Hentzer M, Molin S, Kjelleberg S, Rasmussen TB. 2002. Inhibition of quorum sensing in Pseudomonas aeruginosa biofilm bacteria by a halogenated furanone compound. Microbiology 148:87–102.

3. Fong J, Yuan M, Jakobsen TH, Mortensen KT, Delos Santos MMS, Chua SL, Yang L, Tan CH, Nielsen TE, Givskov M. 2017. Disulfide Bond-Containing Ajoene Analogues As Novel Quorum Sensing Inhibitors of Pseudomonas aeruginosa. J Med Chem 60:215–227.

4. Hentzer M, Wu H, Andersen JB, Riedel K, Rasmussen TB, Bagge N, Kumar N, Schembri MA, Song Z, Kristoffersen P, Mane M, Costerton JW, Molin S, Eberl L, Steinberg P, Kjelleberg S, Hùiby N, Givskov M. 2003. Attenuation of Pseudomonas aeruginosa virulence by quorum sensing inhibitors. EMBO J 22:3803–3815.

Supplementary figure 1

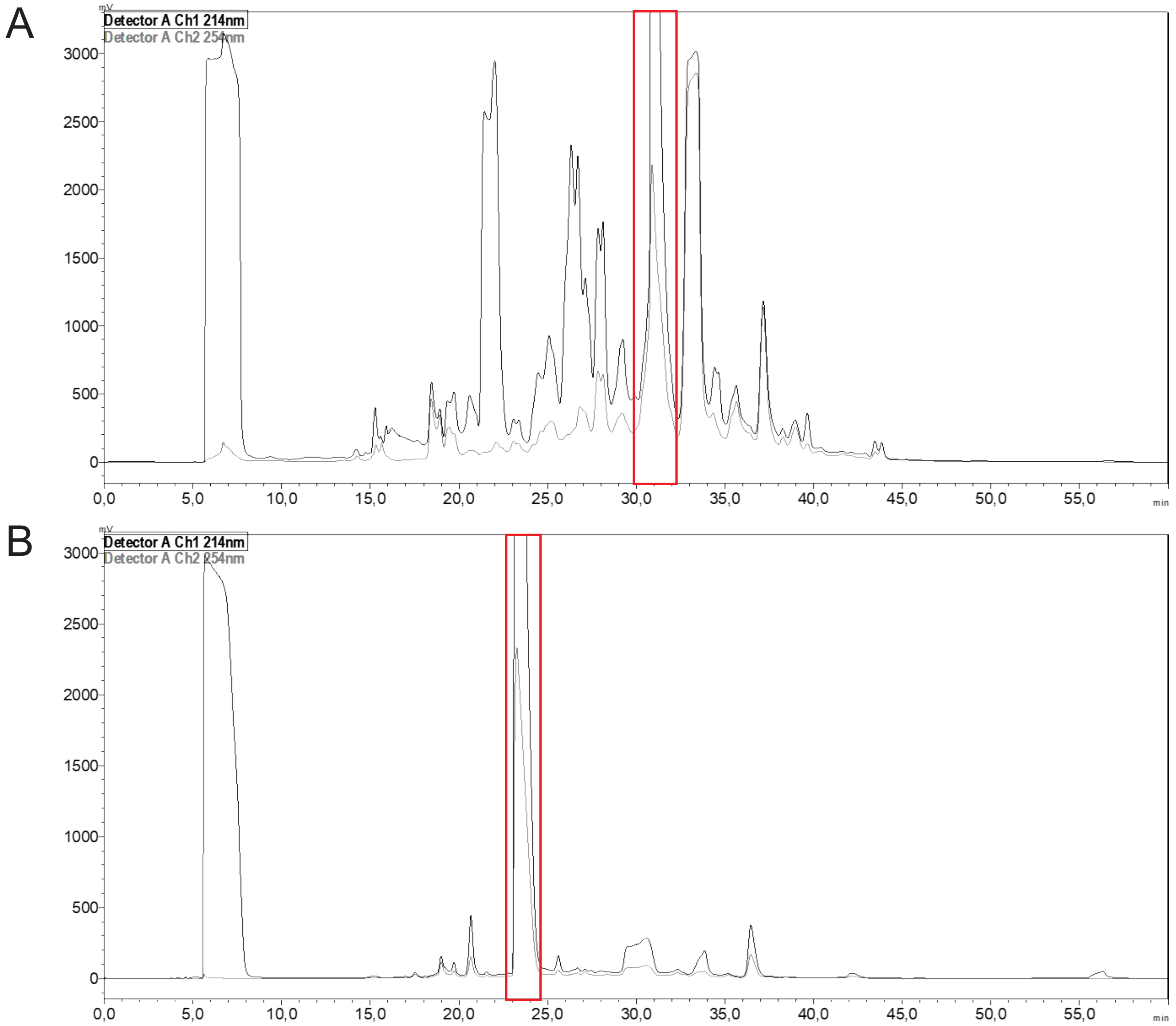

Supplementary Figure 1: Purification of paecilomycone. A) Preparative HPLC spectrogram of *A. allahabadii* supernatant extract using TFA as a modifier, red box outlines the active fraction. B) Subsequent preparative HPLC spectrogram of active fraction using NH_4OAc as a modifier, red box outlines the active fraction that contains paecilomycone.

Supplementary figure 2

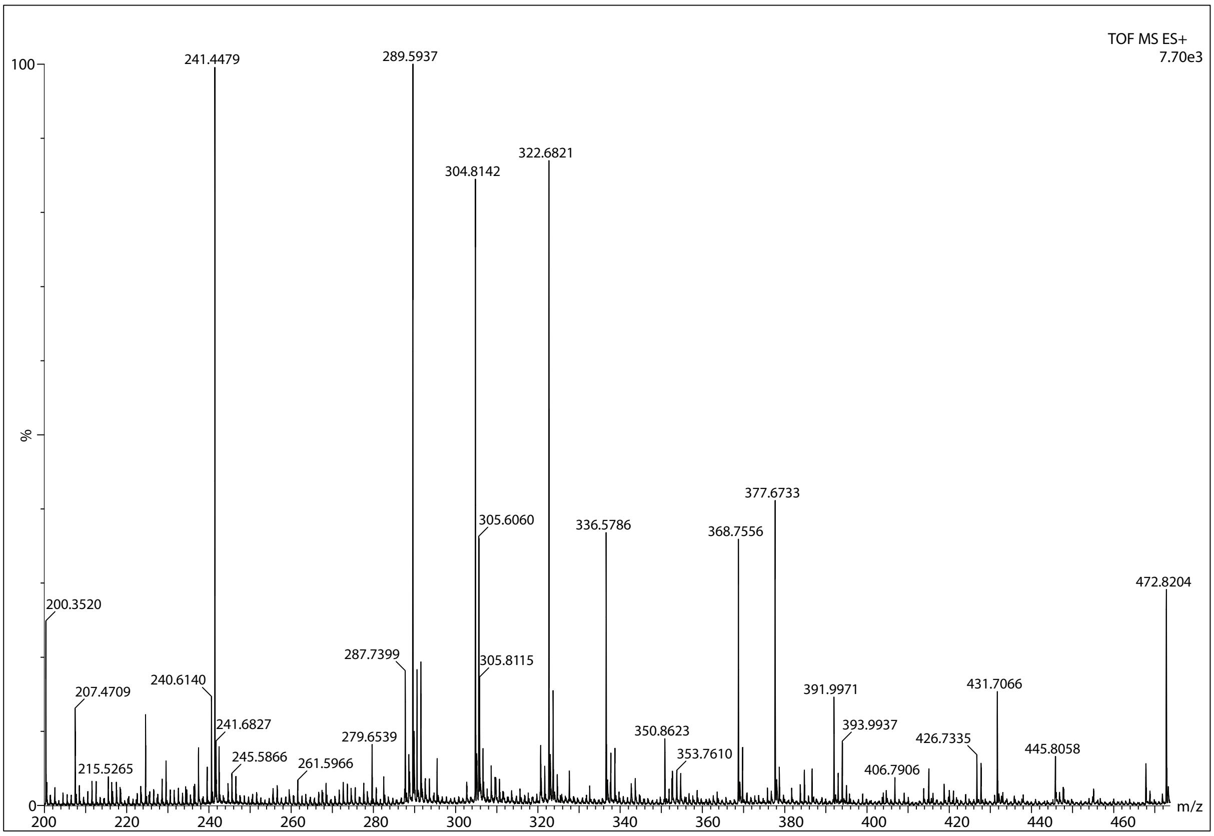

Supplementary Figure 2: High-resolution mass spectrometry chromatogram of paecilomycone A. Indicating a mass of 289.5937.

Supplementary figure 3

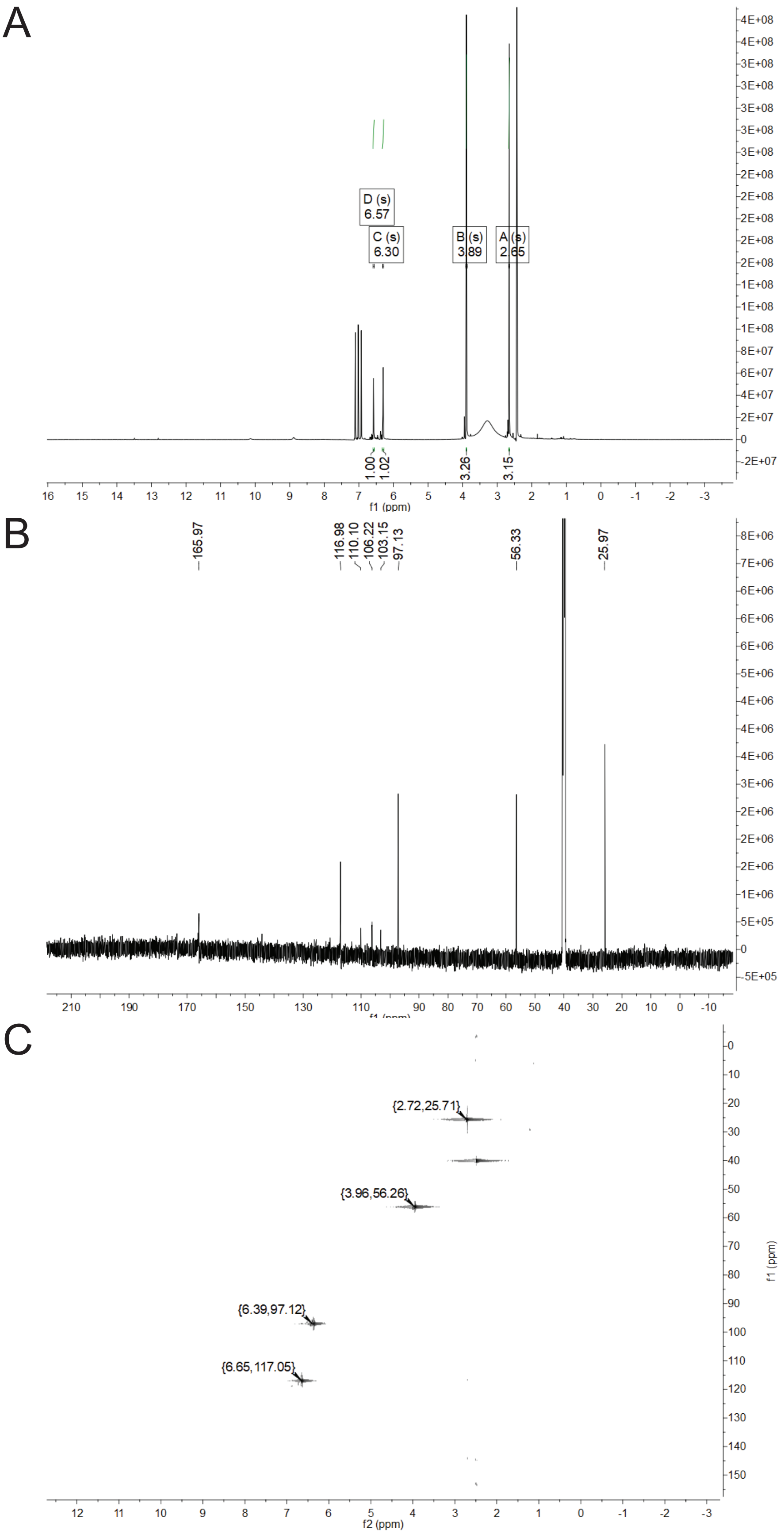

Supplementary Figure 3: NMR data of paecilomycone. A) ^{1}H-NMR of paecilomycone. B) ^{13}C-NMR of paecilomycone. C) 2D-NMR HSQC spectrum

Supplementary figure 4

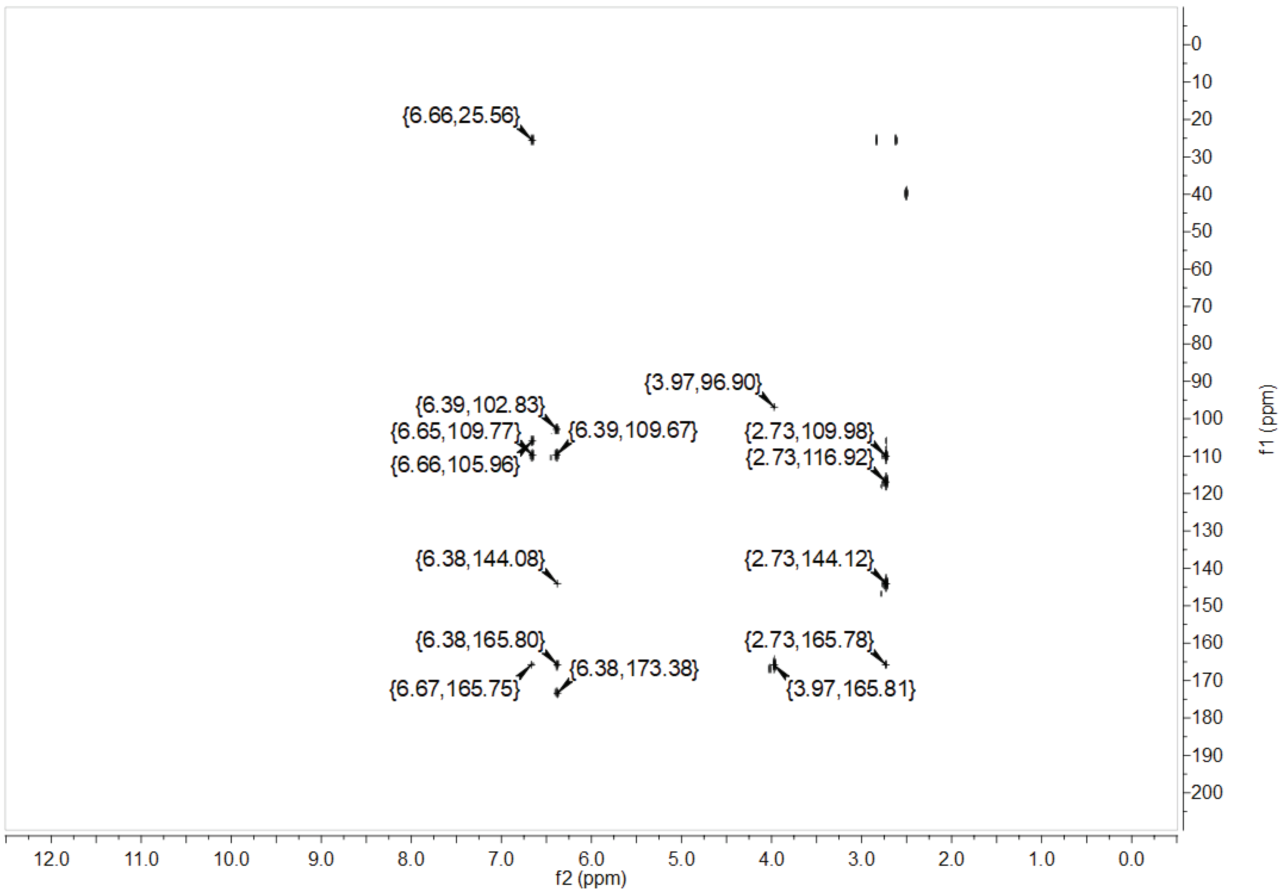

Supplementary Figure 4: 2D-NMR HMBC spectrum

Supplementary figure 5

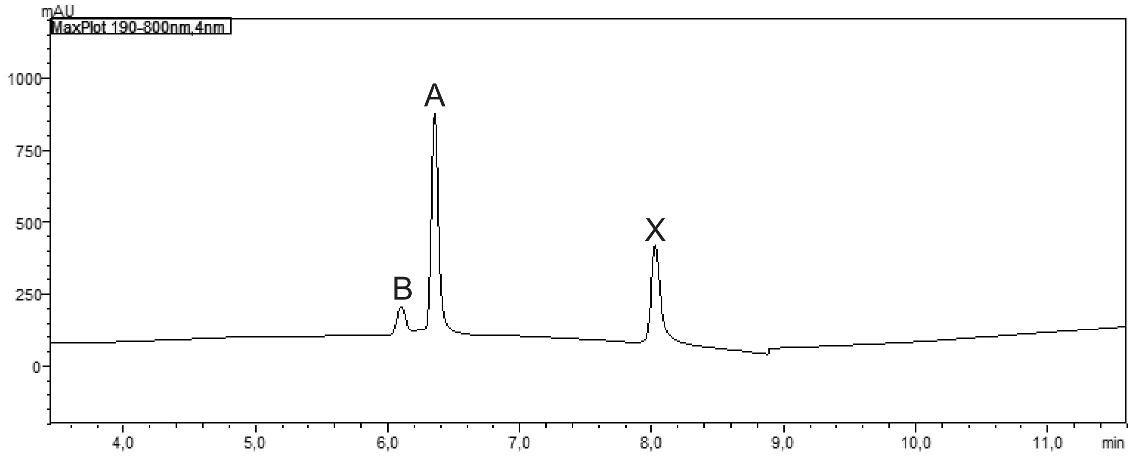

Supplementary Figure 5: aHPLC spectrogram of paecilomycone A without paecilomycone C. This purified fraction does still contain paecilomycone B and an uncharacterized peak

Supplementary figure 6

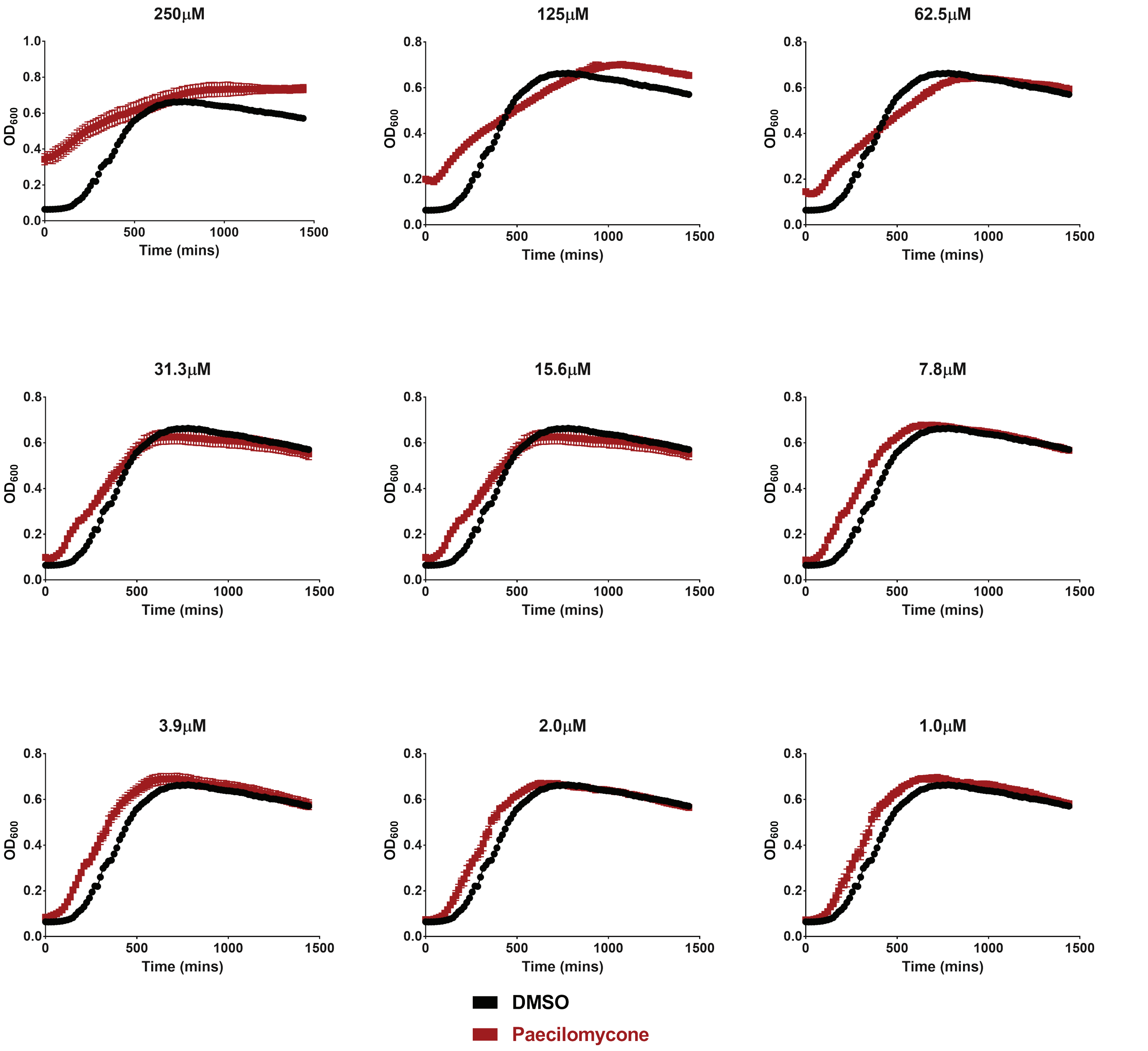

Supplementary Figure 6: Growth of *P. aeruginosa* PAO1 after treatment of paecilomycone in AB medium at 37 °C. Graphs show the growth of 1% DMSO treated (black) versus paecilomycone treated (red). Paecilomycone could interfere with OD_{600} and therefore the start OD_{600} is higher at high concentrations of paecilomycone. Concentrations of paecilomycone used are above the graphs. The experiment was done in triplicates, error bars represent SEM.

Supplementary figure 7

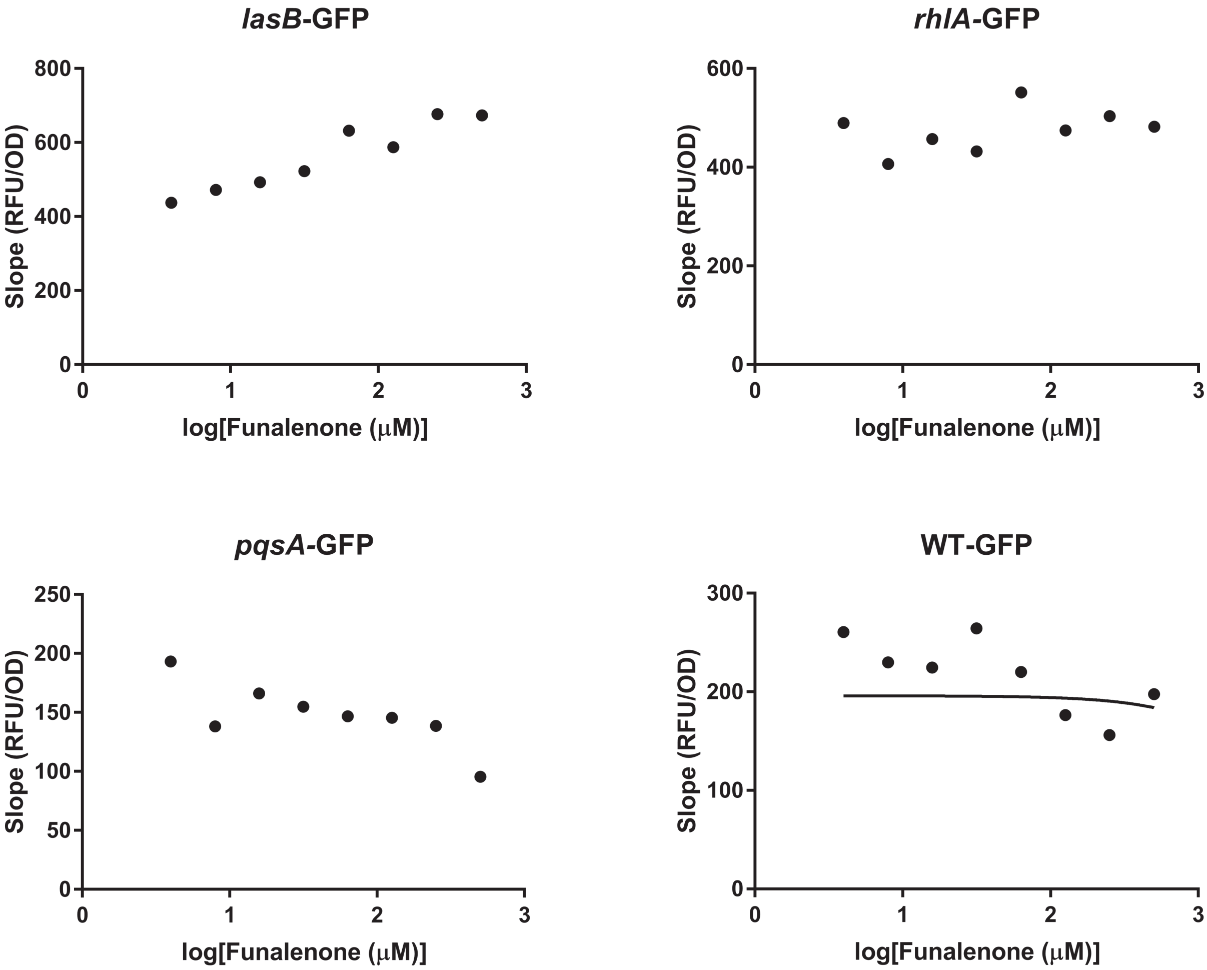

Supplementary Figure 7: Quorum sensing inhibition in *P. aeruginosa* PAO1 reporter strains after funalenone treatment. The effect of funalenone was tested using *lasB*-GFP, *rhlA*-GFP, *pqsA*-GFP reporters. In addition, the effect on WT-GFP as control was tested. The maximum slope of RFU, normalized by growth, was plotted and used to calculate the IC_{50}. Experiments were done three times in triplicates, the mean of RFU/OD is of a representable experiment was plotted in this figure.

Supplementary figure 8

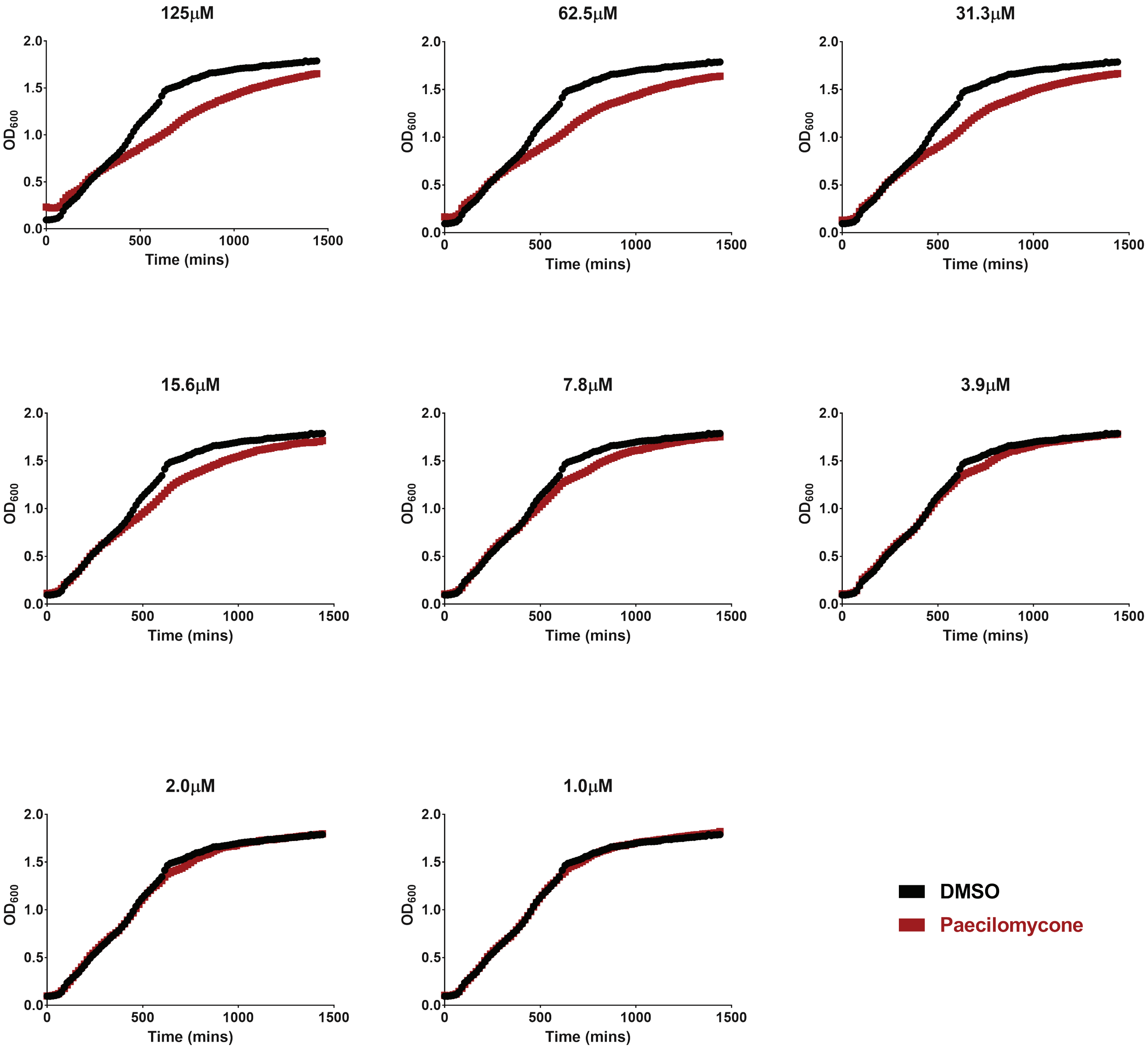

Supplementary Figure 8: Growth of *P. aeruginosa* PAO1 after treatment of paecilomycone in King's A medium at 37 °C. Graphs show the growth of 1% DMSO treated (black) versus paecilomycone treated (red). Paecilomycone could interfere with OD_{600} and therefore the start OD_{600} is higher at high concentrations of paecilomycone. Concentrations of paecilomycone used are above the graphs. The experiment was done in triplicates, error bars represent SEM.

Supplementary figure 9

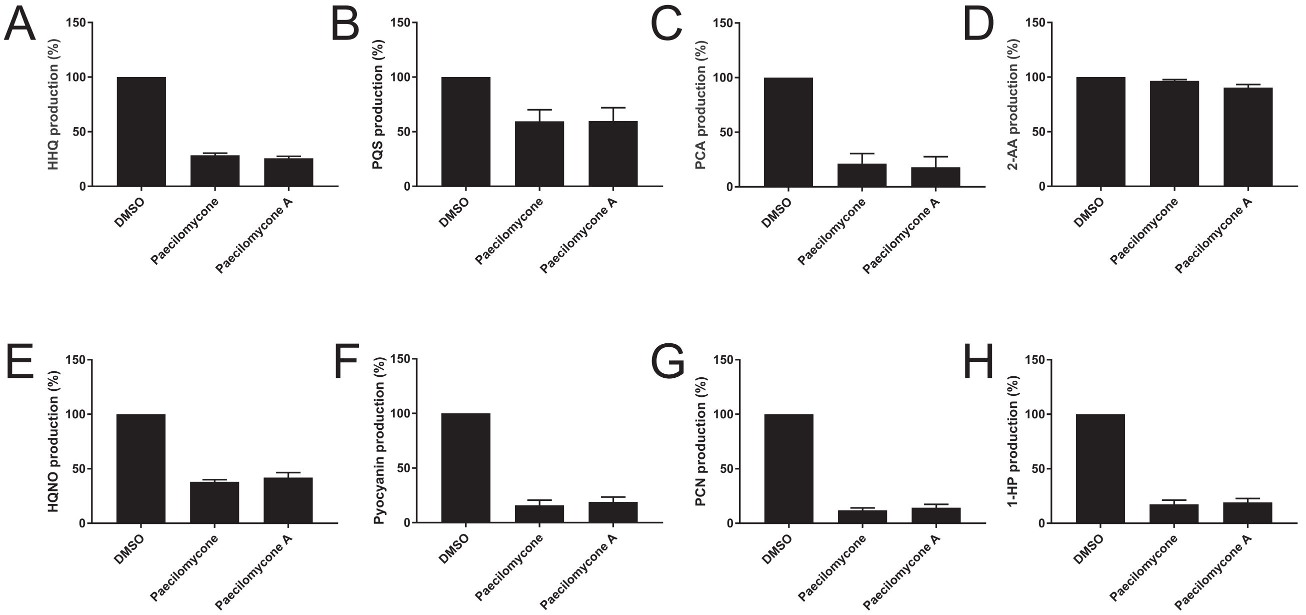

Supplementary Figure 9: Comparison between the effect of paecilomycone and paecilomycone A in the production of various metabolites. Graphs show the production of A) HHQ, B) PQS, C) PCA, D) 2-AA, E) HQNO, F) pyocyanin, G) PCN, H) 1-HP. Experiments were done three times in triplicates, values were normalized to DMSO treated control, and the mean of the experiments is plotted with error bars representing the SEM. Unpaired t-9tests were performed to determine if there is a statistical difference between paecilomycone and paecilomycone A treatment. No statistical difference was found.
